# Evaluating model generalizability for suicide attempt risk prediction: traditional machine vs deep learning

**DOI:** 10.1038/s44184-026-00209-2

**Published:** 2026-04-30

**Authors:** Nicholas Josselyn, Sahil Sawant, Rachel E. Davis-Martin, Elke A. Rundensteiner, Ben S. Gerber, Bo Wang, Anthony J. Rothschild, Emmanuel Agu, Edwin D. Boudreaux, Feifan Liu

**Affiliations:** 1 Data Science, Worcester Polytechnic Institute, Worcester, MA USA; 2 University of Massachusetts Chan Medical School, Worcester, MA USA; 3 Exeliq Consulting Inc., Schaumburg, IL USA; 4 Computer Science, Worcester Polytechnic Institute, Worcester, MA USA

**Keywords:** Computational biology and bioinformatics, Health care, Mathematics and computing, Medical research

## Abstract

Suicide remains a leading cause of death and a significant public health concern in the United States. A majority (83%) of suicide decedents had a healthcare visit within the prior 365 days, presenting unique opportunities to utilize healthcare data for AI-based interventions. While previous works applied machine learning (ML) to analyze healthcare records for suicide attempt risk prediction (SARP), they lack external validation. Additionally, advantages of deep learning (DL) over ML for tabular SARP remains understudied. We performed external validation of a state-of-the-art SARP model from the Mental Health Research Network using over 750,000 UMass Memorial Health patient encounters. We further compared ML vs DL, assessing cross-setting healthcare generalizability. We found existing models did not generalize well, ML significantly outperformed DL on most metrics, and DL achieved higher sensitivity. These findings underscore the need for developing robust, generalizable SARP models for diverse healthcare contexts, improving identification of individuals at risk.

## Introduction

In 2023, a staggering 49,000 individuals lost their lives to suicide in the United States, corresponding to ~1 death every 11 minutes. Further, ~1.5 million adults attempted suicide and 12.8 million adults seriously considered it^1^. Notably, 83% of suicide decedents received healthcare in the year prior to death, yet half did not have a mental health diagnosis, and only 24% had such a diagnosis in the 4-weeks prior to death^2–4^. Improving the ability to identify individuals at high risk for suicide within the healthcare system is an important first step for suicide prevention and addressing this public health concern^5^.

Electronic health record (EHR) databases commonly store healthcare data in tabular format^6–8^. For suicide detection tasks, healthcare data includes tabular features such as demographic, prescription, insurance, and depression screening information. This data represents a mix of numeric and categorical data (captured as sparse one-hot encodings)^9^. For tabular data, tree-based models such as Random Forest and XGBoost^10^ are typically models of choice. Some studies show that traditional machine learning (ML) can outperform deep learning (DL) approaches^11–13^ on tabular data. However, the comparative advantages of these approaches across different datasets remain inconclusive^11–13^.

While recent ML models have demonstrated promising discrimination for suicide attempt risk prediction (SARP) within development populations^7,8,14–19^, a critical limitation of this literature is the near-absence of rigorous external validation. The majority of high-performing models, including those by Dhaubhadel et al.^20^ and Martinez et al.^21^, were developed and validated exclusively within VA populations using internal validation methods. The few studies that have conducted external validation in underrepresented populations^17,18^ reveal both the feasibility and necessity of such testing, demonstrating that model performance can vary substantially when applied to new contexts. This generalizability gap represents a major barrier to clinical implementation, as models that perform well in development cohorts may fail to maintain accuracy when deployed in diverse real-world settings^22^. To promote nationwide implementation of data-driven SARP as a core component of healthcare services^23^, it is imperative to better understand existing models’ generalizability, i.e., how well a model developed in one context performs in another.

Additionally, many DL models for SARP utilize non-medical data, such as social media data^24–26^, with some DL works that use Veterans Affairs (VA) EHR data^20,21^. However, the use of DL for tabular data remains limited and sometimes controversial^11–13^. We acknowledge that there is work related to suicide attempt risk prediction outside tabular EHR data, such as using unstructured clinical notes^27–29^, however in this study we focus on structured data analysis. Therefore, we identify a dual need to (1) rigorously evaluate the generalizability of SARP models across different clinical settings, and (2) systematically compare the strengths and limitations of ML versus DL methods for this type of data in the context of suicide attempt risk prediction.

Consequently, in this work, we investigate both ML and DL models for SARP on hospital patient data within 90 days after an outpatient encounter. We study the transferability of models pre-trained on datasets from the Mental Health Research Network (MHRN)^8^ to our UMass Memorial Health (UMMH) dataset, as well as the generalizability of the MHRN model re-trained using UMMH data across different clinical settings. We compare ML and DL models for a variety of transfer tasks across four metrics using UMMH data with over 750,000 patient encounters. We study the generalizability of models across two clinical settings: primary care (PC) vs mental health specialty (MH). Four tasks are defined depending on which clinical setting is used for training and which for testing. Finally, we determine top contributing features for prediction and how well top-performing models perform on sub-groups of race, ethnicity, and sex.

## Methods

We collect data from the UMass Memorial Health System (UMMH) and follow previously outlined collection standards set by the Mental Health Research Network. We then experiment with both ML and DL models. We use ML models such as logistic regression, XGBoost, and Random Forest. We use deep learning models such as simple multi-layer perceptron (MLP), ResNet, and deep tabular learning models FT-Transformer, TabNet, and TabNet variant with unsupervised pre-training. Then we try an ensemble of all models. With these models, we study the generalizability of them under different clinical settings for SARP within 90 days post-physician visit.

**Fig. 1:**
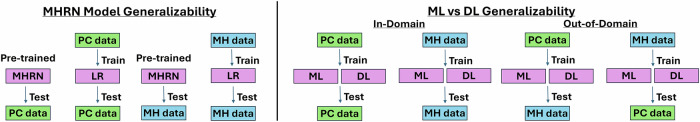
Study Design. Left: MHRN generalizabilty study comparing pre-trained logistic regression (LR) coefficients vs training LR models from scratch on our hospital data. Right: Comparing ML and DL models for in-domain and out-of-domain tasks.

**Fig. 2:**
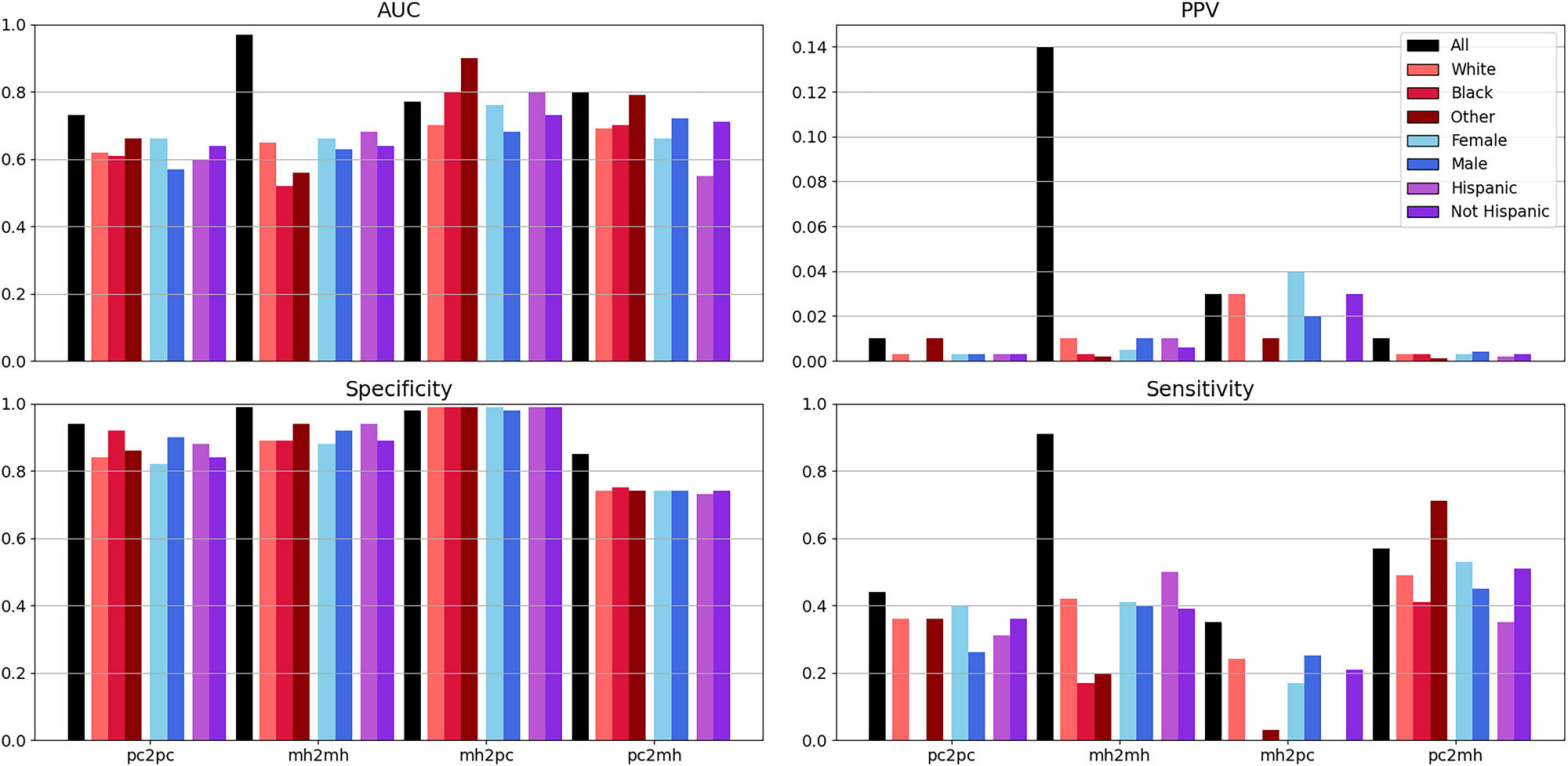
Each plot is a different metric. Each bar is either All data or a sub-group of race (White, Black, Other), sex (Male, Female), or ethnicity (Hispanic, Not Hispanic). Along the x-axis are the 4 transfer tasks that group 8 bars in each.

We evaluate models across four metrics: area under the receiver operating characteristic curve (AUC-ROC), positive predictive value (PPV), specificity, and sensitivity. We define different healthcare setting transfer tasks and introduce our two core experimental studies: (1) the transferability of a pre-trained model^8^ to our large-scale UMMH dataset (on the left side of Fig. 1), and (2) a comparative study of ML vs DL for different transfer tasks (on the right side of Fig. 1), including fairness and feature importance analyses. We release our code and trained models: https://github.com/njosselyn13/SARP_MODEL_GENERALIZABILITY.

### Dataset

We adopt the retrospective data collection protocol from the NIMH-funded Mental Health Research Network (MHRN) study^8^. We create a cohort of patients aged 13 or older who had a primary care encounter (index) between October 1, 2017 and January 21, 2025 at UMMH, and had a mental health diagnosis on the index encounter or on their active problem list. Following MHRN protocol, extracted features include demographic characteristics (age, sex, race, ethnicity, insurance, neighborhood income, and education level), current and past mental health and substance use diagnoses, past suicide attempts, other past injury or poisoning diagnoses, prescriptions for mental health medication, past inpatient or emergency department mental health care, general medical diagnoses (Charlson Comorbidity Index^30^ categories), and recorded scores on the PHQ-9^31^ (total score and item 9 score). A complete list and descriptions are in our GitHub repository.

Using the MHRN protocol, our study cohort contains 755,322 outpatient visits; 321,556 primary care (PC) and 433,766 mental health specialty care (MH). In total, 1141 (0.15%) visits were followed by a suicide attempt within 90 days. Dataset statistics are in Table 1. This retrospective study has been approved by the Institutional Review Board (IRB) of the UMass Chan Medical School (IRB ID: “STUDY00000804”; study title: “Automated, Data-driven, AdaPtable, and Transferable learning (ADAPT) for suicide risk prediction”). The IRB also approved the waiver of authorization for this study (STUDY00000804) under HIPAA, because it meets the “minimum necessary” criteria but it is not feasible to obtain tens of thousands of patients’ authorizations for this retrospective study (no intervention or interactions with the subjects will be done). Following previous work^7,8^, we use patient encounter level data and predict the binary task of a suicide attempt within 90 days given the features defined above. Suicide risk 90-days post visit has been studied in several prior works^7,17–19^, including by MHRN^8^. A suicide attempt is ascertained by a list of ICD-9 or ICD-10 codes defined by MHRN^8^, indicating intentional self-harm. The positive class is a patient that had a suicide attempt within 90 days, and the negative class is a patient that did not.

**Table 1:** Characteristics of primary-care and mental-health specialty datasets

	Primary care				Mental health specialty care			
Characteristic	Total	Train	Valid	Test	Total	Train	Valid	Test
Visits	321,556	135,857	73,154	112,545	433,766	183,265	98,682	151,819
Sex								
Female	198,637	83,817	45,327	69,493	274,875	116,104	62,589	96,182
Male	122,919	52,040	27,827	43,052	158,891	67,161	36,093	55,637
Age group (years)								
13-17	16,070	6734	3682	5654	21,583	9087	4867	7629
18-29	43,489	18,555	9842	15,092	61,207	25,572	13,991	21,644
30-44	66,419	28,021	15,237	23,161	112,386	47,445	25,418	39,523
45-64	110,425	46,440	25,101	38,884	170,317	72,211	38,825	59,281
65+	85,153	36,107	19,292	29,754	68,273	28,950	15,581	23,742
Race								
White	276,188	116,936	62,685	96,567	361,951	152,846	82,510	126,595
Asian	5086	2099	1205	1782	4514	1905	1020	1589
Black	10,358	4,318	2342	3698	17,281	7303	3873	6105
HI/PI	310	125	74	111	523	237	118	168
Native American	1344	583	320	441	2262	970	471	821
Unknown	27,932	11,642	6459	9831	46,011	19,480	10,412	16,119
Ethnicity								
Hispanic	34,610	14,514	8,026	12,070	58,006	24,529	13,135	20,342
Not Hispanic	286,946	121,343	65,128	100,475	375,760	158,736	85,547	131,477
Visits followed by								
Suic. att. w/in 90 days	431	182	98	151	710	300	161	249
Suic. att. w/in 30 days	232	102	54	76	271	103	69	99
Suic. death w/in 90 days	4	3	1	0	39	17	6	16
Suic. death w/in 30 days	1	0	1	0	20	9	3	8

*HI* Native Hawaiian, *PI* Pacific Islander, *Suic.* Suicide, *att.* attempt.

Counts are shown for total, training, validation, and test splits.

Our data splitting is reproducible with a fixed random seed of 42. To reduce the risk of overfitting under severe class imbalance, we split PC and MH data separately into training, validation, and testing sets of size 42.25%, 22.75%, and 35%, respectively. Data splitting is stratified at the patient encounter level. Suicide attempts were rare, resulting in substantial class imbalance. We did not construct balanced datasets via under- or over-sampling; instead, we applied cost-sensitive learning through class weighting, which has been shown to outperform resampling strategies for imbalanced clinical prediction tasks while preserving the underlying data distribution^32^. Data release is pending approval. Additional details are in the supplementary material and code.

The MHRN patient cohort was collected across seven health systems consisting of over 10 million MH visits and over 9.5 million PC visits. There was a mean age of 46 years old and 62% female. In comparison, our dataset is from the UMMH system, is less than 1 million total patient visits with an average age of 46 years old and 63% female. There was similarly a heavy imbalance between suicide attempts within 90 days after visit; MHRN having 0.44% suicide attempts out of all visits. Our UMMH dataset was also more heavily dominated by white patient visits (84.5%) and less for black patient visits (3.7%) than MHRN white patients (67.2%) and black patients (8.6%). A complete dataset statistics table for MHRN is found in their paper^8^.

### Machine learning

First, we validate the MHRN group’s solution on our UMMH data. We experiment with logistic regression (LR) for binary classification. Specifically, we use a LR model that learns from scratch on our data and a LR model that uses the released, pre-trained MHRN coefficients to directly evaluate on our data. LR is a generalized linear model that is simple to implement and obtains interpretable predictions. In LR, feature coefficients are learned via Maximum Likelihood Estimation (MLE). These coefficients are what are released by the MHRN group and used as the pre-trained coefficients in this work.

We then use tree-based ML models, such as XGBoost^10^ and Random Forest that have been shown to work well for tabular data. They are simpler and sometimes superior to DL^11,13,33^. XGBoost is an ensemble of weak learner decision trees where each sequential model aims to correct mistakes made by previous models. Random Forest is another tree-based ensemble learning technique. It uses bagging (bootstrap aggregating) to reduce resultant model variance. In this work, we use the scikit-learn implementations of both.

### Deep learning

In this work we use four DL models. These include two standard DL approaches, multi-layer perceptron (MLP) and Residual Network (ResNet), and two tabular DL models, FT-Transformer and TabNet.

MLP is a feedforward neural network composed of fully connected (dense) neurons and nonlinear activation functions. It has been used as a baseline and simple DL network for tabular data to compare against tree-based and other proposed, more complex DL tabular models (i.e. transformer-based models)^33–36^.

ResNet is a deep learning architecture that utilizes skip connections to allow for information to bypass one or more layers^37^. Traditionally, ResNet uses convolutional layers, however, for tabular data convolutional layers are replaced with fully connected layers while preserving skip connections. In a lot of tabular DL works ResNet is not used as a baseline, however, in ref. ^33^ the authors argue that it is a strong baseline competitive with tree-based models, MLPs, and proposed transformer-based models.

FT-Transformer is a DL model with transformer backbone that embeds each numerical and categorical feature into a dense vector space^33^. It then applies a transformer backbone composed of multi-head attention and feed forward networks across the feature tokens. FT-Transformer’s self-attention captures higher order feature dependencies. Typically, FT-Transformer performs well for tabular datasets with diverse feature types (continuous and categorical). In our UMMH dataset, we have a large number of both, with a majority of categorical (binary) features.

TabNet is a DL model with a transformer backbone designed for tabular data^38^. It enables end-to-end learning with minimal feature engineering. Inspired by decision trees, TabNet performs sparse, instance-wise feature selection, processes data in sequential decision steps, and achieves an ensemble-like effect through high-dimensional representations.

TabNet’s encoder consists of feature transformer blocks, an attentive transformer, masking, and aggregation. The feature transformer splits into two paths: one for final aggregation and one to the attentive transformer, which generates sparse masks to select relevant features at each decision step. These selected features pass through step-specific transformers, and the outputs are aggregated for prediction. The decoder, built from similar blocks, enables unsupervised pretraining by reconstructing masked features. A pretraining ratio indicates the proportion of features to reconstruct, allowing the model to learn from large, unlabeled datasets and improving performance when labels are limited.

### Ensemble learning

To combine the predictions of heterogeneous base learners, we employed a stacking ensemble with logistic regression as the meta-classifier. First, each base model (XGBoost, Random Forest, TabNet variants, FT-Transformer, MLP, and ResNet) was independently evaluated on a held-out validation set to generate predicted probabilities for the positive class. These probabilities were then concatenated to form a meta-feature matrix, where for each patient the positive class validation set probability for each model is stored. A logistic regression model was trained on this validation-derived meta-feature space using the corresponding ground-truth labels, learning an optimal linear combination of base model outputs. At test time, the trained meta-model was applied to the stacked predicted probabilities from the test set to produce final ensemble predictions.

### Transfer tasks

Transfer tasks refer to the healthcare setting (PC or MH) a model is trained vs tested on. We define two types: in-domain (top of Table 3) and out-of-domain (bottom of Table 3) and can be seen in Fig. 1. The source domain is the training data; the target domain is the test data. *In-domain* refers to training and testing being done on the same domain. These tasks include training and testing on PC only (pc2pc) or MH only (mh2mh). *Out-of-domain* refers to training on one domain and testing on another. These tasks include training on PC and testing on MH (pc2mh) and training on MH and testing on PC (mh2pc).

### MHRN model generalizability study design

Our first study explores the generalizability of released models by the MHRN group^8^ to our UMMH dataset. MHRN trained and tested LR models on PC or MH data only. Their released LR coefficients are used as pre-trained coefficients in our LR model, when applicable. These can be found at: https://github.com/MHResearchNetwork/srpm-model. MHRN releases non-zero coefficients, identified using LASSO, for 102 of 320 features for their PC data and 94 for their MH data. The remaining 218 and 226, respectively, are zero. All 320 features match the 320 we have in our dataset.

We define three scenarios to train and evaluate LR models for PC and MH settings (see Table 2). First, we use the 102 (PC) or 94 (MH) released coefficients to predict a suicide attempt on our UMMH data, i.e. no training. Second, LR models are trained from scratch on our data, but only using the 102 (PC) or 94 (MH) features identified by MHRN. Lastly, LR models are trained from scratch on our data using all 320 features. Training with and without class balancing is done. All LR models trained from scratch use Optuna^39^ hyperparameter tuning optimized for maximum AUC on the validation set of data. Hyperparameters such as regularization, optimization tolerance, and solver choice are tuned. All hyperparameter ranges searched are in our released code.

**Table 2:** Logistic regression (LR) for pc2pc (top) and mh2mh (bottom) tasks

Domain	Model	# Feats.	Balancing	AUC	PPV	Specificity	Sensitivity
PC	Pre-trained (MHRN)	102	–	0.67	0.03	0.99	0.25
	LR	102	No	**0.76**	**0.78**	**1.0**	0.09
		102	Yes	**0.76**	0.01	0.88	**0.55**
	LR	320	No	**0.76**	**0.78**	**1.0**	0.09
		320	Yes	0.72	0.01	0.90	0.52
MH	Pre-trained (MHRN)	94	–	0.65	0.01	0.99	0.03
	LR	94	No	0.82	0.63	**1.0**	0.02
		94	Yes	0.89	0.01	0.84	0.76
	LR	320	No	0.89	**0.71**	**1.0**	0.05
		320	Yes	**0.92**	0.01	0.89	**0.88**

Pre-trained vs trained from scratch on 102 (PC), 94 (MH), or 320 features, with or without class balancing. Highest values in bold for each metric for PC (top) and MH (bottom).

### ML vs DL generalizability study design

We compare ML and DL models for in-domain and out-of-domain transfer tasks. Results are in Table 3 with mean and bootstrapped (1000 random samples) 95% confidence intervals (subscripts) on a test set described in the Dataset Section. Using models introduced in the Methods Section, we compare ML models XGBoost and Random Forest to the DL models MLP, ResNet^37^, FT-Transformer^33^, and TabNet and its variant with unsupervised pre-training^38^. Models are optimized for AUC using Optuna^39^, with a max of 100 epochs, 100 training trials sampling hyperparameters, class weight balancing where available, and using all 320 features. Key hyperparameters such as learning rate and weight decay are tuned, with all hyperparameter ranges searched are in our released code.

**Table 3:** In-domain (top) and out-of-domain (bottom) results

	Model	Source	Target	AUC	PPV	Specificity	Sensitivity
In-domain	XGBoost	PC	PC	0.80_[0.75, 0.84]_	0.47_[0.36, 0.59]_	1.0_[1.0, 1.0]_	0.22_[0.15, 0.29]_
		MH	MH	0.98_[0.96, 0.99]_	0.99_[0.97, 1.0]_	1.0_[1.0, 1.0]_	0.70_[0.64, 0.76]_
	RF	PC	PC	0.77_[0.72, 0.81]_	0.83_[0.69, 0.96]_	1.0_[1.0, 1.0]_	0.17_[0.11, 0.23]_
		MH	MH	0.98_[0.97, 0.99]_	0.94_[0.91, 0.97]_	1.0_[1.0, 1.0]_	0.82_[0.78, 0.87]_
	MLP	PC	PC	0.64_[0.58, 0.70]_	0.33_[0.25, 0.41]_	0.99_[0.99, 1.0]_	0.23_[0.17, 0.31]_
		MH	MH	0.95_[0.93, 0.97]_	0.92_[0.88, 0.96]_	0.99_[0.99, 1.0]_	0.61_[0.55, 0.68]_
	ResNet	PC	PC	0.62_[0.56, 0.68]_	0.37_[0.27, 0.48]_	0.99_[0.99, 1.0]_	0.18_[0.12, 0.25]_
		MH	MH	0.95_[0.92, 0.97]_	0.84_[0.79, 0.88]_	0.99_[0.99, 1.0]_	0.78_[0.72, 0.83]_
	FT-Trans.	PC	PC	0.71_[0.65, 0.76]_	0.57_[0.44, 0.71]_	0.99_[0.99, 1.0]_	0.17_[0.11, 0.23]_
		MH	MH	0.97_[0.95, 0.98]_	0.76_[0.72, 0.81]_	0.99_[0.99, 1.0]_	0.74_[0.68, 0.80]_
	TabNet-0	PC	PC	0.75_[0.70, 0.80]_	0.05_[0.04, 0.06]_	0.99_[0.99, 0.99]_	0.34_[0.26, 0.41]_
		MH	MH	0.97_[0.95, 0.98]_	0.16_[0.15, 0.17]_	0.99_[0.99, 0.99]_	0.90_[0.86, 0.93]_
	TabNet-1	PC	PC	0.73_[0.67, 0.78]_	0.01_[0.01, 0.01]_	0.94_[0.94, 0.94]_	0.44_[0.36, 0.52]_
		MH	MH	0.97_[0.96, 0.99]_	0.14_[0.14, 0.15]_	0.99_[0.99, 0.99]_	0.91_[0.88, 0.94]_
	TabNet-2	PC	PC	0.71_[0.65, 0.76]_	0.05_[0.04, 0.06]_	0.99_[0.99, 0.99]_	0.36_[0.28, 0.44]_
		MH	MH	0.97_[0.95, 0.98]_	0.12_[0.11, 0.12]_	0.99_[0.99, 0.99]_	0.91_[0.88, 0.94]_
	Ensemble	PC	PC	0.75_[0.70, 0.80]_	0.77_[0.66, 0.89]_	0.99_[0.99, 1.0]_	0.23_[0.17, 0.30]_
		MH	MH	0.98_[0.97, 0.99]_	0.99_[0.97, 1.0]_	0.99_[0.99, 1.0]_	0.86_[0.81, 0.90]_
Out-of-domain	XGBoost	MH	PC	0.79_[0.75, 0.83]_	0.0_[0.0, 0.0]_	1.0_[1.0, 1.0]_	0.0_[0.0, 0.0]_
		PC	MH	0.81_[0.77, 0.84]_	0.10_[0.03, 0.17]_	1.0_[1.0, 1.0]_	0.02_[0.01, 0.04]_
	RF	MH	PC	0.77_[0.72, 0.81]_	0.03_[0.02, 0.03]_	0.98_[0.98, 0.98]_	0.35_[0.27, 0.42]_
		PC	MH	0.80_[0.77, 0.84]_	0.02_[0.02, 0.02]_	0.96_[0.96, 0.96]_	0.46_[0.39, 0.52]_
	MLP	MH	PC	0.36_[0.31, 0.42]_	0.14_[0.0, 0.50]_	0.99_[0.99, 1.0]_	0.01_[0.0, 0.02]_
		PC	MH	0.64_[0.60, 0.68]_	0.33_[0.24, 0.41]_	0.99_[0.99, 1.0]_	0.12_[0.08, 0.16]_
	ResNet	MH	PC	0.46_[0.40, 0.52]_	0.06_[0.0, 0.16]_	0.99_[0.99, 1.0]_	0.01_[0.0, 0.03]_
		PC	MH	0.64_[0.60, 0.68]_	0.07_[0.03, 0.11]_	0.99_[0.99, 1.0]_	0.04_[0.02, 0.06]_
	FT-Trans.	MH	PC	0.65_[0.60, 0.70]_	0.15_[0.08, 0.24]_	0.99_[0.99, 1.0]_	0.07_[0.03, 0.12]_
		PC	MH	0.74_[0.71, 0.78]_	0.16_[0.06, 0.29]_	0.99_[0.99, 1.0]_	0.02_[0.01, 0.04]_
	TabNet-0	MH	PC	0.71_[0.67, 0.76]_	0.04_[0.02, 0.05]_	1.0_[1.0, 1.0]_	0.12_[0.07, 0.18]_
		PC	MH	0.80_[0.76, 0.83]_	0.01_[0.01, 0.01]_	0.85_[0.84, 0.85]_	0.57_[0.51, 0.63]_
	TabNet-1	MH	PC	0.68_[0.64, 0.73]_	0.02_[0.01, 0.04]_	1.0_[1.0, 1.0]_	0.05_[0.02, 0.09]_
		PC	MH	0.73_[0.69, 0.76]_	0.01_[0.01, 0.01]_	0.90_[0.90, 0.90]_	0.45_[0.39, 0.52]_
	TabNet-2	MH	PC	0.67_[0.62, 0.72]_	0.02_[0.01, 0.02]_	0.98_[0.98, 0.99]_	0.18_[0.12, 0.24]_
		PC	MH	0.72_[0.68, 0.75]_	0.01_[0.0, 0.01]_	0.93_[0.92, 0.93]_	0.27_[0.22, 0.33]_
	Ensemble	MH	PC	0.77_[0.73, 0.81]_	–	0.99_[0.99, 1.0]_	0.0_[0.0, 0.0]_
		PC	MH	0.83_[0.80, 0.86]_	–	0.99_[0.99, 1.0]_	0.01_[0.0, 0.02]_

Mean values reported over 1000 bootstrapped samples with 95% CI in subscripts.

*TabNet-0* no pre-training, *TabNet-1* pre-trained on 1 domain, *TabNet-2* pre-trained on both domains, *RF* Random Forest, – no positives predicted.

Table 3 depicts three TabNet variants: TabNet-0, TabNet-1, and TabNet-2. TabNet-0 is TabNet without any pre-training. TabNet-1 is TabNet with pre-training on one domain of data (PC or MH). For TabNet-1, the single pre-training domain is the same for training the TabNet classifier (source data). For example, for TabNet-1 pc2mh task, the model pre-training and classifier are trained on PC data only, and then evaluated on MH data. TabNet-2 is TabNet with pre-training on both domains of data (PC and MH). We tune the pre-training ratio for TabNet with values of 0.2, 0.5, and 0.8. The larger the ratio, the more difficult the pre-training task; a ratio of 0.5 works best.

### Fairness study for subgroups

We then investigate model performance on sub-groups of race (white, black, other), ethnicity (Hispanic, not Hispanic), and sex (male, female). An area of growing interest surrounding how to appropriately deploy SARP models^40,41^ We identify best performing models for each task based on sensitivity, resulting in using TabNet-1 for pc2pc and mh2mh, TabNet-0 for pc2mh, and Random Forest for mh2pc. We base our choice on sensitivity because in general PPV is low, while specificity is high. When choosing based on AUC, we observed a larger sacrifice in sensitivity. Since sensitivity measures the number of true positive suicide attempts, this is important to maximize. Additional model selection details are in the Supplementary material with sensitivity vs AUC performance shown in Supplementary Tables 10, 11.

We assess all models in Fig. 2 on the held-out test set, do bootstrap sampling, and calculate demographic parity (DPR) and equalized odds ratios (EOR) as fairness metrics. DPR is the ratio of the predicted positive outcome rates between groups. EOR is the ratio of the true positive rates between groups. Ratios of 1 are considered fair, with a four-fifths rule (0.80) as generally acceptable. Hawaiian/Pacific Islander, Native American, and Asian had the least representation in the PC and MH test sets. Therefore, we grouped these three, with unknown race, to one group called “other”.

### Feature importance

We analyze top-performing ML (XGBoost) and DL (TabNet-1) models to determine which features are most impactful for prediction using SHapley Additive exPlanations (SHAP)^42^. Across a majority of tasks and metrics, for ML models, XGBoost tends to outperform Random Forest. For DL models, TabNet-1 is one of the models that tends to outperform the other DL models. For ML and DL, the models are trained on the same domain as the target as these task configurations yield the best performances. We identify the top 10 features assessed on PC and MH data using mean SHAP value. We then compare how these identified features using SHAP compare to the important features identified by MHRN using LASSO.

### Evaluation metrics

We use four standard metrics: area under the receiver operator characteristic curve (AUC-ROC), positive predictive value (PPV), specificity, and sensitivity. Each uses true positives (TP), false positives (FP), true negatives (TN), and false negatives (FN). AUC was computed in a threshold-independent manner using predicted probabilities. PPV, sensitivity, and specificity were computed using the default classification threshold (0.5). Details on each metric are provided in the Supplementary material.

In our fairness analysis, we use two fairness metrics: demographic parity ratio (DPR) and equalized odds ratio (EOR). DPR is the ratio of the predicted positive outcome rates between groups. EOR is the ratio of the true positive rates between groups.

### Statistical analysis

For every model pair (36 pairs), we calculate two-sided and one-sided (both less and greater than) paired bootstrap resampling significance tests^43–45^. We adapt this test from https://github.com/neubig/util-scripts/blob/master/paired-bootstrap.py. Two-sided tests tell us whether the model pairs are statistically different. The one-sided tests tell us the direction of significance. We determine whether a model is significantly better or worse than another across all four metrics (AUC, PPV, specificity, sensitivity). We use the 1000 paired bootstrap samples for each metric to compute the paired bootstrap tests. This approach appropriately accounts for the resampling structure of bootstrap data, does not assume normality of the metric distributions, and has been used in prior comparative model evaluation studies^43,44^. A *p*-value <0.05 is considered significant. Complete tables of all *p*-values are in the Supplementary Tables 1–8, significant findings are summarized in the Results.

## Results

### MHRN model generalizability

Results for the pre-trained MHRN model vs training from scratch study are in Table 2. The top of the table are results for PC data, the bottom for MH data. In general, training from scratch outperforms using the pre-trained MHRN model by a large margin, demonstrating its poor generalizability. When training from scratch on UMMH data, balancing during training improves sensitivity and the sensitivity-specificity trade-off by a large margin. This is important because identifying true suicide attempts is a critical task. It is interesting to note that when training from scratch, there is little difference across metrics when using 102 features for PC and 94 features for MH vs all 320 features. This confirms MHRN’s results suggesting that less features can be used to achieve similar performance, offering insights into feature selection being valuable as a pre-processing step.

For PC data, we achieve increases of: 9% in AUC, 75% in PPV, 1% in specificity, and 30% in sensitivity by training from scratch compared to using pre-trained MHRN coefficients. For MH data, we achieve increases of: 27% in AUC, 70% in PPV, 1% in specificity, and 85% in sensitivity by training from scratch. *These large increases by training on hospital-specific data highlight that models from one hospital are not generalizable to data from another, even when the same features are extracted. This further motivates the necessity of developing more advanced, generalizable models and critically evaluating them across hospital settings*.

### ML vs DL generalizability

Results for ML and DL models for all transfer tasks are in Table 3. ML generally outperforms DL on average, but DL provides better sensitivity, which is crucial for identifying as many true suicide attempts as possible given the serious public health consequences of missed cases. Developing strong DL models for tabular data is important as it enables future multi-modal models^38,46,47^ that can incorporate rich amounts of image, text, and tabular data for effective diagnosis.

For pc2pc, XGBoost has the highest AUC (0.80) with significant improvement over DL models and the ensemble (*p*-value < 0.001 or <0.01). Random Forest (0.77) is a close second and FT-Transformer, the ensemble, and all TabNet approaches are over 0.70. Random Forest has the highest PPV (0.83) with the next highest, the ensemble (0.77), meaning Random Forest is confident in its positive predictions. However, given the limited number of positive cases, it is not surprising that generally PPV is low. XGBoost and Random Forest are tied for highest specificity (1.0) with all others being high. Even though ML models are slightly higher, the DL TabNet models are very close for specificity and offer a better specificity-sensitivity trade-off. This means all models do well at identifying patients not at risk, i.e., they are not overdiagnosing. Finally, TabNet-1 has the highest sensitivity (0.44) and significantly outperforms ML, ensemble, and other DL models. This means TabNet is better at performing the critical task of identifying patients truly at risk of an attempt.

For mh2mh, XGBoost, Random Forest, and the ensemble have the highest AUC (0.98). With the ensemble being the most consistently statistically significantly better than other models. All TabNet variants and FT-Transformer are close behind (0.97). Similar to pc2pc, ML outperforms DL, but DL is close behind with a better specificity-sensitivity trade-off. XGBoost and the ensemble (0.99) have the highest PPV and are significantly better than all other models (*p*-value < 0.001). Random Forest is close behind (0.94), however MLP (0.92), ResNet (0.84), and FT-Transformer (0.76) achieve competitive performance. All offer high specificity (0.99–1.0). However, TabNet-1, TabNet-2 (0.91), and TabNet-0 (0.90) offer significantly superior sensitivity (*p*-value < 0.001).

*Overall, for in-domain, ML has the highest AUC, PPV, and specificity; DL has superior sensitivity*. TabNet achieves a better specificity-sensitivity trade-off while maintaining competitive AUC. Higher sensitivity is crucial because more true suicide attempts are correctly identified. We note mh2mh tasks have overall higher values than pc2pc, potentially due to having more suicide attempts and overall data from MH visits.

For pc2mh, the ensemble has a significantly higher AUC (0.83) than all other models (*p*-value < 0.001, <0.01, or <0.05). XGBoost (0.81), Random Forest (0.80) and TabNet-0 (0.80) are close behind, with all tabular DL models over 0.70. MLP has a significantly higher (*p*-value < 0.001 or <0.05) PPV (0.33) than all other models, with FT-Transformer coming in second (0.16). The ensemble is unable to predict any positive cases and thus has no recorded PPV. XGBoost has the highest specificity (1.0), with all models still reporting high values. Again, TabNet, specifically TabNet-0 (0.57), has a significantly higher (*p*-value < 0.001 or <0.01) sensitivity than all other models.

For mh2pc, XGBoost has the highest AUC (0.79) and is significantly higher (*p*-value < 0.001 or <0.05) than all other models, except the ensemble. Random Forest, the ensemble, and TabNet-0 have AUC also over 0.70. Interestingly, FT-Transformer has a significantly higher PPV (0.15) than most other models, but, all models are generally low (0.0–0.15). The ensemble is unable to predict any positive cases and thus has no recorded PPV. All models have high specificity (0.98–1.0). Random Forest has the highest sensitivity (0.35) and is significantly higher (*p*-value < 0.001) than TabNet-2 (0.18), the second highest. This differs from the general trend of DL outperforming ML on sensitivity. PPV and sensitivity are generally lower for out-of-domain tasks, meaning it is harder to identify true positives, and the PC-MH domain gap is large enough to cause confusion.

Overall, out-of-domain tasks have similar conclusions as in-domain. ML is better for most metrics, while DL has better sensitivity – but only for pc2mh. For mh2pc, ML outperforms DL for sensitivity, but it is low for all models in this task. *This domain gap in out-of-domain tasks suggests the importance of training and evaluating on the same domain, with generalizability across healthcare settings remaining a desired goal*.

PC target tasks are tested on the PC domain; training is done on PC or MH. XGBoost, trained on PC, provides the highest AUC (0.80). Random Forest, trained on PC, has the highest PPV (0.83). For specificity, there are five instances of 1.0 trained on PC or MH, with all over 0.90. TabNet-1, trained on PC, provides the highest sensitivity (0.44). This shows similar trends that ML outperforms DL on most metrics, but DL has better sensitivity. Further, it reinforces that *training with data from one visit type does not generalize well when evaluating on a different type*.

MH target tasks are tested on the MH domain; training is done on PC or MH. XGBoost, Random Forest, and the ensemble trained on MH have the highest AUC (0.98), but all TabNet models and FT-Transformer trained on MH are 0.97. XGBoost and the ensemble (0.99) trained on MH offer superior PPV. All models trained on MH have specificity of 0.99 or 1.0, with 8 out of 9 models trained on PC over 0.90. TabNet-1 and TabNet-2 trained on MH offer the highest sensitivity (0.91), with TabNet-0 trained on MH close behind (0.90). *Again, we observe training with data from one domain does not generalize well to another*.

### Fairness of models for subgroups

In Fig. 2, we show results for the best performing model, based on sensitivity as explained in the Methods section, for all tasks and metrics for race, ethnicity, and sex groups compared to the total ("All”) population. Additional tables are in Supplementary Tables 12–18. In general, PPV is low, specificity is high, and the mh2mh task for all patients has the highest metric values.

For race specificity, there is not much disparity in identifying patients without a suicide attempt between white and black patients. However, for PPV and sensitivity, there is a larger disparity in identifying suicide attempts. In some cases PPV and sensitivity are zero for black patients. It is important to note that for PC target there is only one black patient with a suicide attempt and twelve for MH. This unfair treatment to black patients agrees with existing ML for suicide detection work^40^. Specific demographic data statistics for the test set are broken down in Supplementary Table 9. Race DPR and EOR is less than 0.60 for all models, except pc2mh (DPR = 0.97). The generally adopted four-fifths rule would suggest these are unfair. For ethnicity, the model’s performance on the Hispanic group is lower on all metrics, especially sensitivity. DPR and EOR are less than 0.80 for all models, except pc2mh (DPR = 0.97), showing potential ethnicity bias. However, pc2pc DPR and EOR is 0.79 and close to fair. For sex, DPR and EOR are less than 0.70, except pc2mh again (DPR = 0.99, EOR = 0.85). The CDC reported in 2023 male suicide rates were approximately four times higher than females, and males made up ~80% of suicides^1^. Our data has more female representation and more PC and MH females with suicide attempts. All DPR and EOR values are shown in Supplementary Table 19.

### Feature importance analysis

**Fig. 3:**
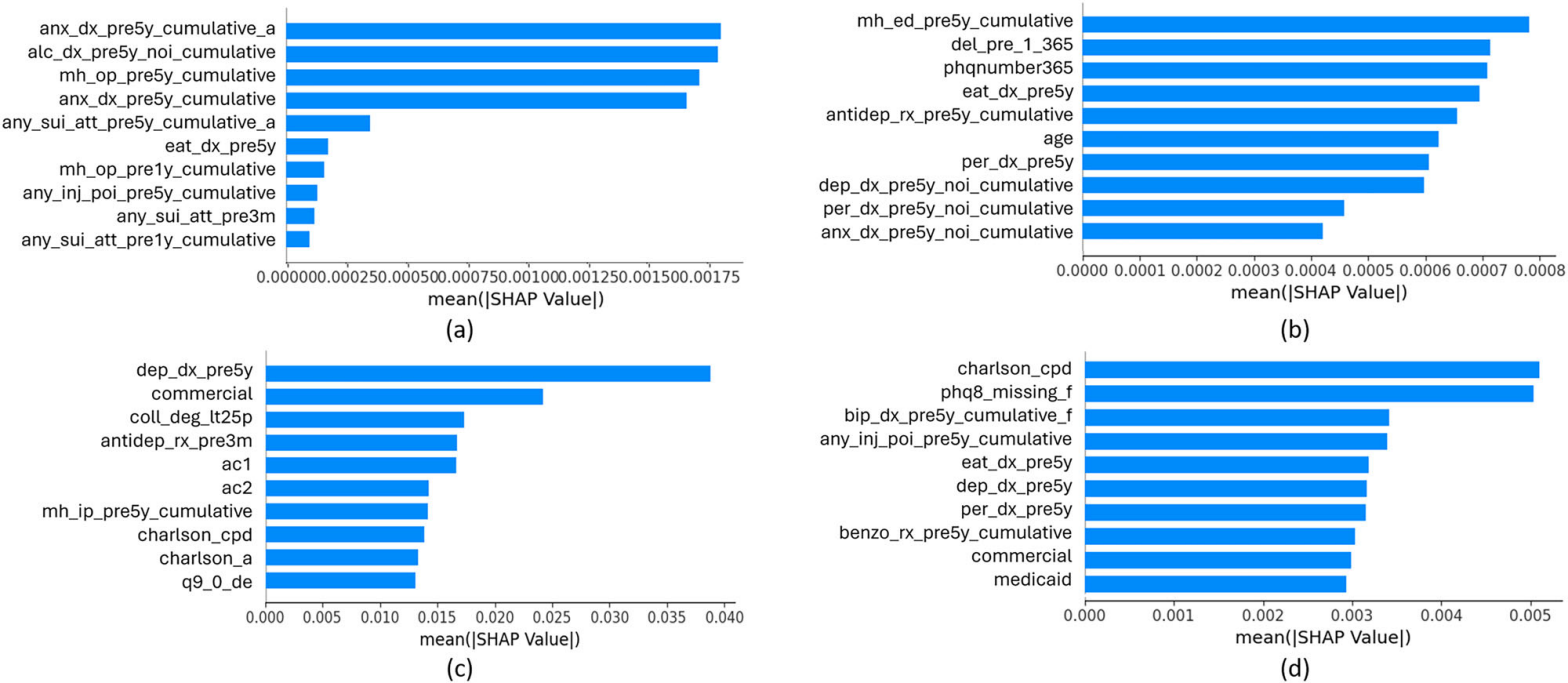
SHAP analysis. **a** XGBoost (ML) PC Target, **b** XGBoost (ML) MH Target, **c** TabNet-1 (DL) PC Target, **d** TabNet-1 (DL) MH Target Top 10 features for each on y-axes. Mean of the absolute value of all SHAP values on x-axes. Features related to prescription (rx) drug information, drug/alcohol (alc) use, depression (dep)/anxiety (anx)/bipolar (bip)/eating disorder (eat)/personality disorder (per) diagnoses (dx), previous suicide attempts (sui_att), and age (ac1, ac2) are identified as important. Detailed feature name descriptions in Supplementary Table 22.

SHAP analysis is shown in Fig. 3a, b for the ML XGBoost model with testing on PC and MH, respectively. In Fig. 3c, d, we show SHAP for the DL TabNet-1 model on PC and MH, respectively. These SHAP values do not indicate if the feature helps the model positively or negatively, but overall the impact it has on a prediction. We identify the top ten features for each of the four models. Additional SHAP figures are in the Supplementary Figs. 2, 3.

For both ML and DL models, we identify that features related to prescription drug information, depression/eating/personality disorder diagnoses, mental health hospital visits, age, and PHQ information are important. There are a total of 33 out of 40 unique features identified across all four models. For the same target domain, comparing ML vs DL, there are little to no exact overlapping top 10 features identified. There are some commonalities for PC target domain such as mental health visitation utilization. For MH target domain, both have features related to PHQ information, eating/personality disorder diagnoses; with 2 features exactly overlapping: eating disorder (eat_dx_pre5y) and personality disorder (per_dx_pre5y) diagnoses within the previous 5 years.

Comparing SHAP identified features for each model to MHRN’s 94 MH and 102 PC LASSO-selected important features, we find a subset of our 33 unique features within each. For the 94 MH features, 20 of the 33 features we identify are contained in the 94. For the 102 PC features, 22 of the 33 are contained in the 102. A complete set of these features are shown in Supplementary Table 20. *A majority of our top contributing features are therefore aligned with previous work*. However, it is important to note that feature importance analysis techniques used between our work and MHRN differ (SHAP vs LASSO) and thus can contribute to some of the disagreements. Descriptions of all 33 unique features are provided in Supplementary Table 21.

## Discussion

In this study, we evaluated the generalizability of suicide attempt risk prediction (SARP) models across primary care and mental healthcare settings. Specifically, we first examined machine learning (ML) models released by the National Institute of Mental Health (NIMH)-funded Mental Health Research Network (MHRN) and assessed their performance when applied to our UMass Memorial Health (UMMH) dataset. We then compared the advantages and limitations of ML and deep learning (DL) approaches on our UMMH. We conducted experiments across a range of training and testing configurations, evaluated multiple metrics, and analyzed outcomes across subgroups defined by race, ethnicity, and sex.

*We found that the pre-trained MHRN logistic regression models for SARP were not directly transferable to our UMMH dataset*. This is shown via a large increase in performance when trained on UMMH data as compared to directly utilizing the model trained on MHRN, with matching extracted EHR features. This finding underscores the importance of external validation studies prior to the adoption of any models in clinical practice. Additionally, it highlights the need for diverse and complete sets of training data. Patient populations, clinician workflow patterns, diagnostic coding practices, and other factors may differ across hospitals; thus capturing these shifts between settings is crucial.

Additionally, experiments using the UMMH dataset showed that *ML models generally outperformed DL models, with the exception that DL demonstrated higher sensitivity*. This aligns with prevailing evidence that ML methods tend to perform better on tabular data. However, it is worth pointing out that the DL performance on tabular SARP was comparable on average to ML models across most metrics and the DL models consistently achieved superior sensitivity, a key consideration when identifying patients at risk is critical.

Furthermore, we showed that *models trained using the UMMH datasets do not generalize well across different clinical settings*: primary care and mental health specialty. This underscores the importance of developing SARP models that can generalize well across different healthcare contexts (e.g., different clinical settings, geographic locations, and demographic diversities). For future work, we will explore incorporating advanced transfer learning and domain adaptation techniques to improve the model’s generalizability.

For our generalizability study across primary care and mental health data within the same UMMH dataset, we observed generally high or acceptable AUC values across all transfer tasks and models, along with consistently high specificity. *The most variability emerged in PPV and sensitivity, which are two metrics tied to performance on the positive (suicide attempt) class*.

We observe that for in-domain tasks, XGBoost, Random Forest, MLP, ResNet, FT-Transformer, and the ensemble have average to good PPV values, meaning there aren’t a lot of false positives and the models are identifying at risk patients but not over-predicting. The three lowest metrics arise for the pc2pc tasks (MLP, ResNet, XGBoost). This demonstrates that correctly identifying at risk patients in the PC domain is difficult, potentially due to the smaller sample size of suicide attempts in the PC cohort. The highest PPV values for in-domain tasks occur for XGBoost and the ensemble for the mh2mh task.

For all TabNet models, PPV is very low. We observe that for XGBoost, Random Forest, MLP, ResNet, and FT-Transformer models, the number of true positives and false positives are low for mh2mh. However, there is a sharp increase in false positives for TabNet models while still maintaining comparable true positive values, thus decreasing PPV and over-predicting suicide attempts. Therefore, it is important to consider the excess in costs and burden a low PPV model would incur on a hospital system. To allow for a detailed examination of false positve and true postive trade-offs, the raw confusion matrices can be found in Supplementary Fig. 1.

For out-of-domain tasks, PPV is generally low and consistently lower than in-domain tasks. All out-of-domain PPV values that are 0.10 or higher, the 95% confidence intervals are large. This indicates that even if the mean is slightly higher, it is quite unstable. These low PPV values indicate that models not trained and tested on the same data type are not reliable at identifying the true suicide attempts. These low PPV values are reflective of the overall low true positive values across all model-task pairs. The issue of over-predicting suicide attempts with TabNet models continues in the out-of-domain context. Again, the detailed confusion matrices can be found in Supplementary Fig. 1.

For in-domain tasks, pc2pc sensitivity is lower than mh2mh sensitivity by a large margin. This is again in part due to the higher prevalence of suicide attempts in the mental health cohort of patients. For out-of-domain tasks, sensitivity is lower than in-domain. However, within out-of domain tasks, we observe a slightly better sensitivity performance on MH target tasks in general. We can see that sensitivity, in particular for TabNet models, are generally higher than PPV values from the fact that there are far less false negatives than false positives. This means that even though TabNet models are over-predicting suicide attempts, they are capturing a large portion of patients who are at risk. This is important as this mistake could otherwise have tragic outcomes.

*Our fairness analysis underscores the critical role of clinical heterogeneity in adversely affecting model robustness and the importance of ethical considerations for model deployment*. Consistent with prior literature^40^, our findings emphasize the necessity of incorporating diverse data across race, sex, ethnicity, and other demographic subgroups. We observed notable performance variations among those demographic subgroups: while specificity remained high overall, both PPV and sensitivity were substantially lower for certain groups – in particular for black patients compared to white patients as seen in Fig. 2.

Based on the four-fifths rule, most demographic parity ratio and equalized odds ratio values fell below the ideal threshold (≥0.80) for fairness, with the exception of the pc2mh task, which approached parity. It is important to note, however, that the sample sizes for certain subgroups are relatively small, e.g., only one black patient in the PC cohort and twelve in the MH cohort with a suicide attempt. To develop models that generalize well across diverse patient populations and to accurately assess potential disparate impacts, representative data across demographic subgroups is essential^48^.

*A critical consideration for this work is how these models can be integrated into real clinical workflows and what “deployable” means in a hospital setting*. In practice, such models could be used to support clinicians in identifying patients at elevated risk for suicide attempts, enabling timely and targeted intervention. However, operational deployment faces several challenges.

First, clinicians must ensure transparency, accountability, and respect for patient autonomy. Therefore, model explainability and interpretability are essential to support informed and trustworthy clinical decision-making. It is also critical to ensure that those using the technology are properly trained. Whether they are clinicians using an AI tool or a non-domain expert interpreting results, there needs to be full transparency.

Second, false positives and false negatives must be carefully addressed. High false-negative rates imply missed opportunities to intervene with patients in need, potentially causing harm, whereas high false-positive rates can burden patients and healthcare providers, resulting in unnecessary stress, increased workload, and higher healthcare costs. Managing these trade-offs for machine learning-based SARP models is essential for safe, ethical, and sustainable adoption in clinical practice.

Third, clinical heterogeneity adversely affects machine learning models’ robustness due to many factors, including different EHR vendors, clinical practice variations, care settings, data and concept shifting, and demographic diversities. These complexities emphasize the motivation of our study – examining to what extent an existing ML or DL model of suicide attempt risk prediction could generalize across different healthcare contexts and identifying pros and cons of various approaches.

Given the continued prevalence of suicide and suicide attempts, identifying and intervening with patients engaged in the healthcare system is a critical necessity. Patients attending primary care or mental health specialty visits represent key touchpoints for effective intervention. However, identifying patients at risk of suicide is a complex and challenging task influenced by many factors, such as genetics, personality characteristics (e.g. impulsivity and aggression), childhood trauma, mental and physical illnesses, substance abuse, availability to lethal means, among several others^49^. The analysis of large volumes of patient data and diversity of potential factors naturally motivates the use of AI-powered risk assessment tools. Despite this need, limited work has explored the application of ML and DL models for SARP. Further, the benefits of ML versus DL for tabular data, including electronic health records, remain inconclusive.

*Our study has several limitations*. First, only two clinical settings were considered for generalizability assessment, however these settings were chosen based on the original settings included in the MHRN data and propensity for suicide risk in these settings. Future studies should explore how models could be adapted to more clinical settings. Second, additional post-physician visit timeframes for assessing suicide risk should be investigated such as 7-day and 3–day post-physician visit^19^. Third, we didn’t test the model’s temporal generalizability which will be explored in the future. Fourth, we only explored class-weighting to overcome the data imbalance issue common for clinical prediction tasks. More investigation is needed to employ other techniques to address this challenge, including for example data augmentation or focal loss. And fifth, the integration of unstructured data, including clinical notes, is of interest to further improve model performance^27–29^.

In summary, we conducted comprehensive experiments to assess SARP models’ generalizability across different institutions and clinical settings. Although traditional ML achieved better overall performance than DL models, complementary advantages on different evaluation metrics were observed. Generalizability gaps for both traditional ML and DL models were reported, suggesting the critical need for developing effective transfer learning and domain adaptation techniques to enable adaptive learning towards improved generalizability.

## Supplementary information

Supplementary information

## Data Availability

The datasets generated and/or analyzed during the current study are not publicly available due to patient privacy but are available from the corresponding author on reasonable request.

## Code availability

The underlying code for this study can be accessed via this link: https://github.com/njosselyn13/SARP_MODEL_GENERALIZABILITY.
