# Supplementary material for: Evaluating model generalizability for suicide attempt risk prediction: traditional machine vs deep learning
Source: Npj Ment Health Res. 2026 Apr 30;5:32. doi: 10.1038/s44184-026-00209-2 (PMC13338226; doi:10.1038/s44184-026-00209-2)
Supplement: Supplementary file 1 — Supplementary information [file 44184_2026_209_MOESM1_ESM.pdf]

Evaluating Model Generalizability for Suicide Attempt Risk Prediction: Traditional Machine vs Deep Learning

Nicholas Josselyn[1,2], Sahil Sawant[1,2,3], Rachel E. Davis-Martin[2], Elke A. Rundensteiner[1,4], Ben S. Gerber[2], Bo Wang[2], Anthony J. Rothschild[2], Emmanuel Agu[1,4], Edwin D. Boudreaux[2], Feifan Liu[2*]

[1]Data Science, Worcester Polytechnic Institute, Worcester, 01609, MA, USA.
[2]University of Massachusetts Chan Medical School, Worcester, 01655, MA, USA.
[3]Exeliq Consulting Inc., Schaumburg, 60173, IL, USA.
[4]Computer Science, Worcester Polytechnic Institute, Worcester, 01609, MA, USA.

*Corresponding author(s). E-mail(s): feifan.liu@umassmed.edu;
Contributing authors: njjosselyn@wpi.edu; sssawant@wpi.edu; Rachel.Davis-Martin@umassmed.edu; rundenst@wpi.edu; ben.gerber@umassmed.edu; bo.wang@umassmed.edu; Anthony.Rothschild@umassmemorial.org; emmanuel@wpi.edu; Edwin.Boudreaux@umassmed.edu;

Supplemental Material

Dataset Feature Names

An excel file dictionary can be found in the GitHub repository listing all features in the dataset and corresponding definitions. File name: "Analytic data dictionary original.xlsx"

Data Statistics and Preparation

Here we re-iterate parts from the main manuscript and add some additional details.

Using the MHRN protocol, our study cohort contains 755,322 outpatient visits; composed of 321,556 primary care (PC) and 433,766 mental health specialty care (MH) outpatient visits. In total, 1,141 (0.151%) outpatient visits were followed by a suicide attempt within 90 days. Statistics on demographics and number of suicide attempts and deaths are provided in Table 1 in the main manuscript.

There are 4 possible target variables to predict in the dataset. A suicide attempt within 30 or 90 days are denoted as event30 and event90, respectively. Suicide death within 30 or 90 days are denoted as death30 and death90, respectively.

We adopt the protocol from the NIMH-funded Mental Health Research Network (MHRN) study and create a cohort of patients aged 13 or older who had at least one outpatient visit with recorded mental health diagnoses from October 1, 2017 through January 21, 2025 from our hospital Epic repository.

Extracted features are from up to 5 years before a patient visit. These features include: demographic characteristics (age, sex, race, ethnicity, insurance, and neighborhood income and education level), current and past mental health and substance use diagnoses (12 categories), past suicide attempts, other past injury or poisoning diagnoses, prescriptions for mental health medication (4 categories), past inpatient or emergency department mental health care, general medical diagnoses (by Charlson Comorbidity Index categories), and recorded scores on the PHQ-9 (including total score and item 9 score). Features are represented as binary or numeric.

Each of the 12 diagnosis categories has 3 overlapping indicators: recorded at or within 90 days before visit, recorded within 1 year before, or recorded within 5 years before.

We predict the binary task of any suicide attempt within 90 days and drop the remaining 3 target variables relating to suicide attempts and death from the dataset for training. The positive class is then a patient "at risk" of a suicide attempt and the negative class is a patient "not at risk". Additionally, we drop variables such as primary_care_visit (identifier of a PC or MH visit), person_id (unique patient identifier number), and vist_mh (whether the visit was to a mental hospital or not). In the end there are 320 predictor feature columns.

For all experiments we use reproducible data splitting with a random seed of 42. We split PC and MH data separately into training, validation, and testing sets. We split 35% of PC data to a held-out test set and similarly 35% of MH data. The remaining 65% of each is then split to 35% for PC and 35% for MH validation sets; the remaining 65% for each is used for training. All splitting of data is stratified to maintain a proportional amount of each class for each split. In scenarios where we pretrain on PC and MH together, we concatenate the two training datasets after splitting.

For data pre-processing, we drop extraneous columns outside the 320 MHRN defines. We scale all numeric columns in PC and MH cohorts using sklearn preprocessing scale function. If a column is completely empty, it is dropped. If a column contains any empty values, it is imputed with -1.

Hyperparameter Tuning

Here we re-iterate parts from the main manuscript and add some additional details.

All experiments for all models are tuned and optimized using Optuna. For each task where the training-validation source-target transfer task data domains changes, optimization is done. A maximum of 100 epochs is run and all models are optimized on the AUC-ROC metric. For all experiments and models, class weight balancing is enabled. A consistent set of hyperparameter search space is defined for all experiments and appears in the released codes for training each model.

For TabNet pre-training experiments, the pre-training ratio is tuned. This determines the level of masking for unsupervised reconstruction. We try ratios of 0.2, 0.5, and 0.8. The interpretation of the pre-training ratio is, for example: a pre-training ratio of 0.8 means the model must reconstruct 80% of the features from 20% of the remaining data; the larger the ratio, the more difficult the pre-training task. We determine 0.5 works best and is what we use for all reported results that use pre-training.

We report a mean and bootstrapped 95% confidence interval (in table subscripts) on a held-out test set. One thousand stratified random samples are taken from the test set; each metric is calculated one thousand times, averaged, and a 95% confidence interval is reported.

Evaluation Metrics

We use four standard metrics: area under the receiver operator characteristic curve (AUC-ROC), positive predictive value (PPV), specificity, and sensitivity. Each uses true positives (TP), false positives (FP), true negatives (TN), and false negatives (FN).

PPV, or precision, measures the proportion of predicted positives (suicide attempts) that are actually true positives (TP/(TP+FP)). High PPV means that when the model predicts a positive case, it is usually correct. PPV is highly impacted by the prevalence of positive cases. Since our data is imbalanced with $< 1\%$ of positive suicide attempts, low PPV is expected.

Sensitivity, or recall, measures the proportion of actual positives that are correctly identified by the model (TP/(TP+FN)). High sensitivity means the model is good at identifying patients at risk of a suicide attempt. Sensitivity is a crucial metric because missing a positive case is concerning as it can lead to the death of a patient. Sensitivity is not impacted by positive class prevalence as much as PPV.

Specificity measures the proportion of actual negatives that are correctly identified by the model (TN/(TN+FP)). Having a high specificity is important because we do not want to misdiagnose a person not at risk as at risk of a suicide attempt. This would introduce additional stress and unnecessary treatment to the patient along with additional costs to the healthcare system.

ROC curve is a plot of true positive rate (TPR), or sensitivity, vs false positive rate (FPR=FP/(FP+TN)) under varied decision thresholds. AUC is then the area under the ROC curve and is a measure of the overall model's ability to discriminate between positives and negatives across all possible thresholds. AUC of 1.0 is a perfect classifier and 0.5 is random guessing.

In our fairness analysis, we use two fairness metrics: demographic parity ratio (DPR) and equalized odds ratio (EOR). DPR is the ratio of the predicted positive outcome rates between groups. EOR is the ratio of the true positive rates between groups. Ratios of 1 are considered fair.

Confusion Matrices

In Supplementary Figure 1 we provide a full set of confusion matrices for all 32 model-task pairs. More detailed breakdown of true positives, false positives, true negatives, and false negatives discussed in the main paper can be seen here. For example, as explained in the main paper, the number of false positives for TabNet models sees a sharp increase compared to ML models.

Statistical Significance

Paired Bootstrap Significance Test

Supplementary Tables 1 and 2 reports statistical significances for the pc2pc in-domain task, Supplementary Tables 3 and 4 for mh2mh, Supplementary Tables 5 and 6 for pc2mh, and Supplementary Tables 7 and 8 for mh2pc.

For each model pair, we calculate two-sided and one-sided (both less and greater) paired bootstrap resampling significance tests. Two-sided tests tell us whether the model pairs are statistically different. The one-sided greater than (p (>)) test tells us if Model A is statistically significantly better than Model B. The one-sided less than (p (<)) test tells us is Model A is statistically significantly less than Model B. For p-values: * indicates p-value <0.05; ** indicates p-value <0.01; *** indicates p-value <0.001; NS means not significant.

Fairness

We show here more detailed tables of results for fairness that appear as Figure 1 in the main manuscript, including stats on how many patients per demographic subgroup. Values are the same, but just displayed as tables here. We then explain and show how models were selected based off sensitivity instead of AUC as the "best" models to do our fairness analysis. Finally, we provide a table of fairness metrics.

Demographic Subset Data Stats

In Supplementary Table 9 we report how many patients per demographic subgroup (sex, race, ethnicity) are recorded for the fairness study. These numbers are per group for the test set.

Best Model Selection

We show fairness results using one model we identify as best for each of the 4 tasks. We select the best model based on sensitivity resulting in using the TabNet-1 model for pc2pc and mh2mh tasks, TabNet-0 for pc2mh, and Random Forest for mh2pc.

We base our choice on sensitivity because in general PPV is fairly low, specificity is always high, and when choosing based on AUC there is a larger sacrifice in sensitivity performance for each task-respective model.

We show in Supplementary Tables 10 and 11 the best performing models and their metric values when making model choice based on AUC or sensitivity, respectively.

Fairness Results Tables

Fairness tables for mean and 95% confidence intervals (CI) for race (white, black, other), sex (female, male), and ethnicity (Hispanic, not Hispanic) sub-groups are shown in Supplementary Tables 12–18. These results are shown in Figure 1 in the main manuscript. In that Figure 1 the mean values from these tables are what are shown. These tables provide more detailed information on results.

Fairness Metrics

In Supplementary Table 19 we show demographic parity and equalized odds ratio metrics we refer to in the main text.

Feature Importance

SHAP

We analyze top performing ML (XGBoost) and DL (TabNet-1) models for which subset of features are most impactful for predictions. We do this using the framework SHapley Additive exPlanations (SHAP). We show in the main manuscript in Figure 2 the mean absolute value SHAP values for the top 10 features for each model. Here in Supplementary Figures 2 and 3 we show SHAP beeswarm plots.

SHAP beeswarm plots show the distribution of SHAP values for each feature across all instances. Each dot on the plot is a training data instance. On the x-axis (SHAP value) this represents how much each feature contributes to the model's output. Positive values indicate a positive impact on prediction while negative values indicate a negative impact. Red and blue points relate to the feature value magnitude. Red points represent high feature values, blue low. A lot of features are binary in the dataset and thus have feature values of 0 or 1.

Supplementary Figure 2a shows SHAP analysis for the XGBoost model tested on PC target data and 2b shows SHAP analysis for the XGBoost model tested on MH target data.

Supplementary Figure 3a shows SHAP analysis for the TabNet-1 model tested on PC target data and 3b shows SHAP analysis for the TabNet-1 model tested on MH target data.

Feature Lists

We identify a unique set of 33 features identified using SHAP across the best ML (XGBoost) and DL (TabNet-1) models for both PC and MH targets. In previous work, the MHRN group identified 102 features for PC data and 94 features for MH data

using LASSO that they released coefficients for that we use in our Logistic Regression model comparison. We compare the SHAP features we identify against these. There are 20 out of the 33 unique features we identify that are also found in the 94 MH features and 22 out of 33 that are also found in the 102 PC features. In Supplementary Table 20 we highlight which of the 33 unique features are included and excluded in the PC and MH cohorts.

Descriptions of the 33 unique features identified are explained in Supplementary Table 21.

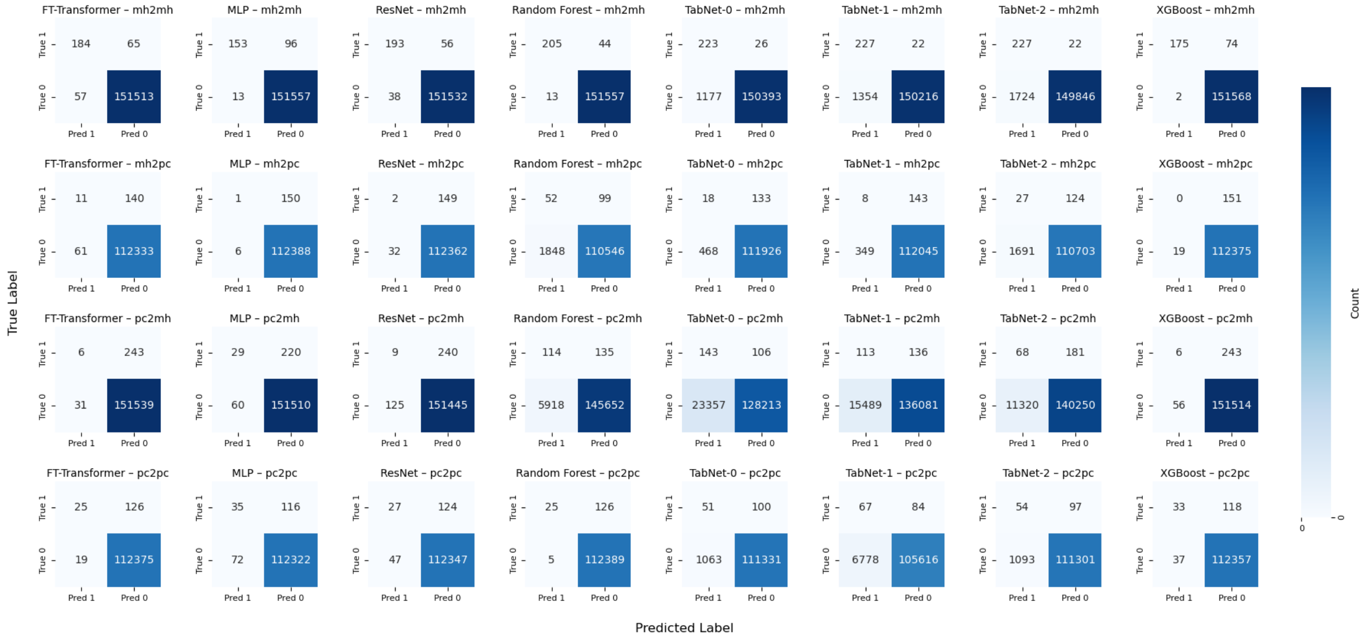

Supplementary Fig. 1: Confusion matrices.

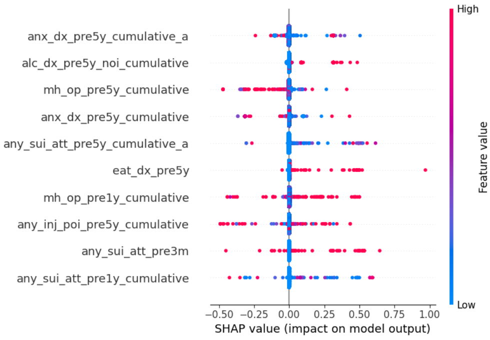

(a) XGBoost (ML) PC Target

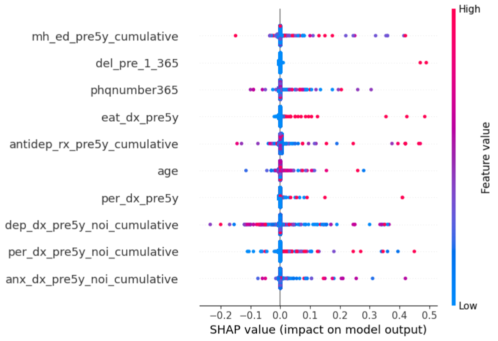

(b) XGBoost (ML) MH Target

Supplementary Fig. 2: SHAP analysis

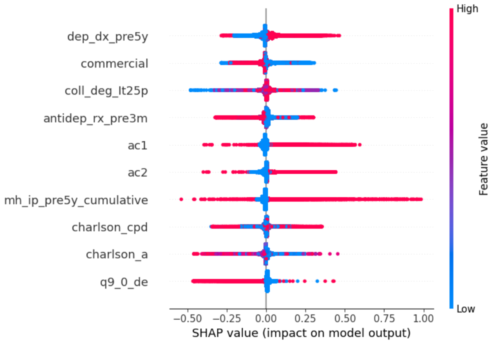

(a) TabNet-1 (DL) PC Target

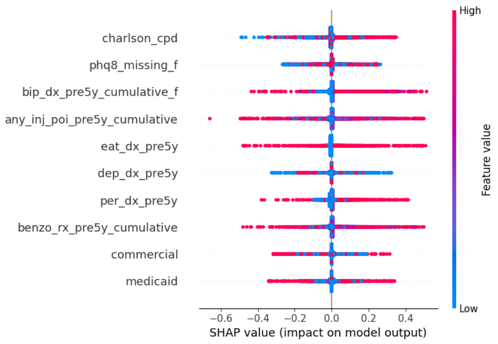

(b) TabNet-1 (DL) MH Target

Supplementary Fig. 3: SHAP analysis

Pairwise Model Comparisons for PC2PC Task – Specificity & Sensitivity

Specificity						Sensitivity					
**Task**	**Model A**	**Model B**	**p**	**p (>)**	**p (<)**	**Task**	**Model A**	**Model B**	**p**	**p (>)**	**p (<)**
PC2PC	TabNet-0	TabNet-2	NS	NS	NS		TabNet-0	TabNet-2	NS	NS	NS
	TabNet-0	MLP	***	NS	***		TabNet-0	MLP	**	**	NS
	TabNet-0	Ensemble	***	NS	***		TabNet-0	Ensemble	***	***	NS
	TabNet-0	FTT	***	NS	***		TabNet-0	FTT	***	***	NS
	TabNet-0	TabNet-1	***	***	NS		TabNet-0	TabNet-1	***	NS	***
	TabNet-0	XGBoost	***	NS	***		TabNet-0	XGBoost	***	***	NS
	TabNet-0	RF	***	NS	***		TabNet-0	RF	***	***	NS
	TabNet-0	ResNet	***	NS	***		TabNet-0	ResNet	***	***	NS
	TabNet-2	MLP	***	NS	***		TabNet-2	MLP	***	***	NS
	TabNet-2	Ensemble	***	NS	***		TabNet-2	Ensemble	***	***	NS
	TabNet-2	FTT	***	NS	***		TabNet-2	FTT	***	***	NS
	TabNet-2	TabNet-1	***	***	NS		TabNet-2	TabNet-1	**	NS	**
	TabNet-2	XGBoost	***	NS	***		TabNet-2	XGBoost	***	***	NS
	TabNet-2	RF	***	NS	***		TabNet-2	RF	***	***	NS
	TabNet-2	ResNet	***	NS	***		TabNet-2	ResNet	***	***	NS
	MLP	Ensemble	***	NS	***		MLP	Ensemble	NS	NS	NS
	MLP	FTT	***	NS	***		MLP	FTT	**	*	NS
	MLP	TabNet-1	***	***	NS		MLP	TabNet-1	***	NS	***
	MLP	XGBoost	***	NS	***		MLP	XGBoost	NS	NS	NS
	MLP	RF	***	NS	***		MLP	RF	**	**	NS
	MLP	ResNet	*	NS	**		MLP	ResNet	*	**	NS
	Ensemble	FTT	*	*	NS		Ensemble	FTT	***	***	NS
	Ensemble	TabNet-1	***	***	NS		Ensemble	TabNet-1	***	NS	***
	Ensemble	XGBoost	***	***	NS		Ensemble	XGBoost	NS	NS	NS
	Ensemble	RF	NS	NS	NS		Ensemble	RF	**	**	NS
	Ensemble	ResNet	***	***	NS		Ensemble	ResNet	*	*	NS
	FTT	TabNet-1	***	***	NS		FTT	TabNet-1	***	NS	***
	FTT	XGBoost	*	**	NS		FTT	XGBoost	**	NS	**
	FTT	RF	**	NS	**		FTT	RF	NS	NS	NS
	FTT	ResNet	***	***	NS		FTT	ResNet	NS	NS	NS
	TabNet-1	XGBoost	***	NS	***		TabNet-1	XGBoost	***	***	NS
	TabNet-1	RF	***	NS	***		TabNet-1	RF	***	***	NS
	TabNet-1	ResNet	***	NS	***		TabNet-1	ResNet	***	***	NS
	XGBoost	RF	***	NS	***		XGBoost	RF	*	*	NS
	XGBoost	ResNet	NS	NS	NS		XGBoost	ResNet	NS	NS	NS
	RF	ResNet	***	***	NS		RF	ResNet	NS	NS	NS

Supplementary Table 1: Bootstrap significance pairwise comparisons between models for Specificity and Sensitivity for the in-domain PC2PC task. * indicates p-value <0.05; ** indicates p-value <0.01; *** indicates p-value <0.001; NS means not significant.

Pairwise Model Comparisons for PC2PC Task – AUC & PPV

AUC						PPV					
**Task**	**Model A**	**Model B**	**p**	**p (>)**	**p (<)**	**Task**	**Model A**	**Model B**	**p**	**p (>)**	**p (<)**
PC2PC	TabNet-0	TabNet-2	NS	NS	NS		TabNet-0	TabNet-2	NS	NS	NS
	TabNet-0	MLP	***	***	NS		TabNet-0	MLP	***	NS	***
	TabNet-0	Ensemble	NS	NS	NS		TabNet-0	Ensemble	***	NS	***
	TabNet-0	FTT	NS	NS	NS		TabNet-0	FTT	***	NS	***
	TabNet-0	TabNet-1	NS	NS	NS		TabNet-0	TabNet-1	***	***	NS
	TabNet-0	XGBoost	*	NS	**		TabNet-0	XGBoost	***	NS	***
	TabNet-0	RF	NS	NS	NS		TabNet-0	RF	***	NS	***
	TabNet-0	ResNet	***	***	NS		TabNet-0	ResNet	***	NS	***
	TabNet-2	MLP	*	*	NS		TabNet-2	MLP	***	NS	***
	TabNet-2	Ensemble	*	NS	*		TabNet-2	Ensemble	***	NS	***
	TabNet-2	FTT	NS	NS	NS		TabNet-2	FTT	***	NS	***
	TabNet-2	TabNet-1	NS	NS	NS		TabNet-2	TabNet-1	***	***	NS
	TabNet-2	XGBoost	***	NS	***		TabNet-2	XGBoost	***	NS	***
	TabNet-2	RF	**	NS	**		TabNet-2	RF	***	NS	***
	TabNet-2	ResNet	**	**	NS		TabNet-2	ResNet	***	NS	***
	MLP	Ensemble	***	NS	***		MLP	Ensemble	***	NS	***
	MLP	FTT	**	NS	**		MLP	FTT	***	NS	***
	MLP	TabNet-1	***	NS	***		MLP	TabNet-1	***	***	NS
	MLP	XGBoost	***	NS	***		MLP	XGBoost	*	NS	**
	MLP	RF	***	NS	***		MLP	RF	***	NS	***
	MLP	ResNet	NS	NS	NS		MLP	ResNet	NS	NS	NS
	Ensemble	FTT	NS	*	NS		Ensemble	FTT	***	***	NS
	Ensemble	TabNet-1	**	**	NS		Ensemble	TabNet-1	***	***	NS
	Ensemble	XGBoost	*	NS	**		Ensemble	XGBoost	***	***	NS
	Ensemble	RF	NS	NS	NS		Ensemble	RF	NS	NS	NS
	Ensemble	ResNet	***	***	NS		Ensemble	ResNet	***	***	NS
	FTT	TabNet-1	NS	NS	NS		FTT	TabNet-1	***	***	NS
	FTT	XGBoost	***	NS	***		FTT	XGBoost	NS	NS	NS
	FTT	RF	*	NS	**		FTT	RF	***	NS	***
	FTT	ResNet	***	***	NS		FTT	ResNet	**	**	NS
	TabNet-1	XGBoost	***	NS	***		TabNet-1	XGBoost	***	NS	***
	TabNet-1	RF	NS	NS	*		TabNet-1	RF	***	NS	***
	TabNet-1	ResNet	***	***	NS		TabNet-1	ResNet	***	NS	***
	XGBoost	RF	NS	NS	NS		XGBoost	RF	***	NS	***
	XGBoost	ResNet	***	***	NS		XGBoost	ResNet	NS	*	NS
	RF	ResNet	***	***	NS		RF	ResNet	***	***	NS

Supplementary Table 2: Bootstrap significance pairwise comparisons between models for AUC and PPV for the in-domain PC2PC task. * indicates p-value <0.05; ** indicates p-value <0.01; *** indicates p-value <0.001; NS means not significant.

Pairwise Model Comparisons for MH2MH Task – Specificity & Sensitivity

	Specificity						Sensitivity					
**Task**	**Model A**	**Model B**	**p**	**p (>)**	**p (<)**	**Task**	**Model A**	**Model B**	**p**	**p (>)**	**p (<)**
MH2MH	MLP	ResNet	***	***	NS		MLP	ResNet	***	NS	***
	MLP	XGBoost	***	NS	***		MLP	XGBoost	**	NS	**
	MLP	TabNet-2	***	***	NS		MLP	TabNet-2	***	NS	***
	MLP	TabNet-0	***	***	NS		MLP	TabNet-0	***	NS	***
	MLP	RF	NS	NS	NS		MLP	RF	***	NS	***
	MLP	FTT	***	***	NS		MLP	FTT	***	NS	***
	MLP	TabNet-1	***	***	NS		MLP	TabNet-1	***	NS	***
	MLP	Ensemble	***	NS	***		MLP	Ensemble	***	NS	***
	ResNet	XGBoost	***	NS	***		ResNet	XGBoost	*	**	NS
	ResNet	TabNet-2	***	***	NS		ResNet	TabNet-2	***	NS	***
	ResNet	TabNet-0	***	***	NS		ResNet	TabNet-0	***	NS	***
	ResNet	RF	***	NS	***		ResNet	RF	NS	NS	*
	ResNet	FTT	*	**	NS		ResNet	FTT	NS	NS	NS
	ResNet	TabNet-1	***	***	NS		ResNet	TabNet-1	***	NS	***
	ResNet	Ensemble	***	NS	***		ResNet	Ensemble	***	NS	***
	XGBoost	TabNet-2	***	***	NS		XGBoost	TabNet-2	***	NS	***
	XGBoost	TabNet-0	***	***	NS		XGBoost	TabNet-0	***	NS	***
	XGBoost	RF	***	***	NS		XGBoost	RF	***	NS	***
	XGBoost	FTT	***	***	NS		XGBoost	FTT	NS	NS	NS
	XGBoost	TabNet-1	***	***	NS		XGBoost	TabNet-1	***	NS	***
	XGBoost	Ensemble	NS	NS	NS		XGBoost	Ensemble	***	NS	***
	TabNet-2	TabNet-0	***	NS	***		TabNet-2	TabNet-0	NS	NS	NS
	TabNet-2	RF	***	NS	***		TabNet-2	RF	***	***	NS
	TabNet-2	FTT	***	NS	***		TabNet-2	FTT	***	***	NS
	TabNet-2	TabNet-1	***	NS	***		TabNet-2	TabNet-1	NS	NS	NS
	TabNet-2	Ensemble	***	NS	***		TabNet-2	Ensemble	***	***	NS
	TabNet-0	RF	***	NS	***		TabNet-0	RF	***	***	NS
	TabNet-0	FTT	***	NS	***		TabNet-0	FTT	***	***	NS
	TabNet-0	TabNet-1	***	***	NS		TabNet-0	TabNet-1	NS	NS	NS
	TabNet-0	Ensemble	***	NS	***		TabNet-0	Ensemble	***	***	NS
	RF	FTT	***	***	NS		RF	FTT	**	**	NS
	RF	TabNet-1	***	***	NS		RF	TabNet-1	***	NS	***
	RF	Ensemble	***	NS	***		RF	Ensemble	*	NS	*
	FTT	TabNet-1	***	***	NS		FTT	TabNet-1	***	NS	***
	FTT	Ensemble	***	NS	***		FTT	Ensemble	***	NS	***
	TabNet-1	Ensemble	***	NS	***		TabNet-1	Ensemble	***	***	NS

Supplementary Table 3: Bootstrap significance pairwise comparisons between models for Specificity and Sensitivity for the in-domain MH2MH task. * indicates $p < 0.05$; ** indicates $p < 0.01$; *** indicates $p < 0.001$; NS means not significant.

Pairwise Model Comparisons for MH2MH Task – AUC & PPV

AUC						PPV					
**Task**	**Model A**	**Model B**	**p**	**p (>)**	**p (<)**	**Task**	**Model A**	**Model B**	**p**	**p (>)**	**p (<)**
MH2MH	MLP	ResNet	NS	NS	NS		MLP	ResNet	***	***	NS
	MLP	XGBoost	**	NS	**		MLP	XGBoost	***	NS	***
	MLP	TabNet-2	*	NS	*		MLP	TabNet-2	***	***	NS
	MLP	TabNet-0	NS	NS	NS		MLP	TabNet-0	***	***	NS
	MLP	RF	**	NS	**		MLP	RF	NS	NS	NS
	MLP	FTT	*	NS	*		MLP	FTT	***	***	NS
	MLP	TabNet-1	NS	NS	*		MLP	TabNet-1	***	***	NS
	MLP	Ensemble	**	NS	**		MLP	Ensemble	***	NS	***
	ResNet	XGBoost	**	NS	**		ResNet	XGBoost	***	NS	***
	ResNet	TabNet-2	*	NS	**		ResNet	TabNet-2	***	***	NS
	ResNet	TabNet-0	*	NS	*		ResNet	TabNet-0	***	***	NS
	ResNet	RF	***	NS	***		ResNet	RF	***	NS	***
	ResNet	FTT	*	NS	**		ResNet	FTT	*	**	NS
	ResNet	TabNet-1	*	NS	**		ResNet	TabNet-1	***	***	NS
	ResNet	Ensemble	***	NS	***		ResNet	Ensemble	***	NS	***
	XGBoost	TabNet-2	NS	NS	NS		XGBoost	TabNet-2	***	***	NS
	XGBoost	TabNet-0	NS	NS	NS		XGBoost	TabNet-0	***	***	NS
	XGBoost	RF	NS	NS	NS		XGBoost	RF	**	**	NS
	XGBoost	FTT	NS	NS	NS		XGBoost	FTT	***	***	NS
	XGBoost	TabNet-1	NS	NS	NS		XGBoost	TabNet-1	***	***	NS
	XGBoost	Ensemble	NS	NS	NS		XGBoost	Ensemble	NS	NS	NS
	TabNet-2	TabNet-0	NS	NS	NS		TabNet-2	TabNet-0	***	NS	***
	TabNet-2	RF	NS	NS	NS		TabNet-2	RF	***	NS	***
	TabNet-2	FTT	NS	NS	NS		TabNet-2	FTT	***	NS	***
	TabNet-2	TabNet-1	NS	NS	NS		TabNet-2	TabNet-1	***	NS	***
	TabNet-2	Ensemble	*	NS	**		TabNet-2	Ensemble	***	NS	***
	TabNet-0	RF	*	NS	**		TabNet-0	RF	***	NS	***
	TabNet-0	FTT	NS	NS	NS		TabNet-0	FTT	***	NS	***
	TabNet-0	TabNet-1	NS	NS	NS		TabNet-0	TabNet-1	***	***	NS
	TabNet-0	Ensemble	***	NS	***		TabNet-0	Ensemble	***	NS	***
	RF	FTT	NS	*	NS		RF	FTT	***	***	NS
	RF	TabNet-1	NS	NS	NS		RF	TabNet-1	***	***	NS
	RF	Ensemble	*	NS	**		RF	Ensemble	***	NS	***
	FTT	TabNet-1	NS	NS	NS		FTT	TabNet-1	***	***	NS
	FTT	Ensemble	**	NS	**		FTT	Ensemble	***	NS	***
	TabNet-1	Ensemble	NS	NS	*		TabNet-1	Ensemble	***	NS	***

Supplementary Table 4: Bootstrap significance pairwise comparisons between models for AUC and PPV for the in-domain MH2MH task. * indicates $p < 0.05$; ** indicates $p < 0.01$; *** indicates $p < 0.001$; NS means not significant.

Pairwise Model Comparisons for PC2MH Task – Specificity & Sensitivity

Specificity						Sensitivity					
**Task**	**Model A**	**Model B**	**p**	**p (>)**	**p (<)**	**Task**	**Model A**	**Model B**	**p**	**p (>)**	**p (<)**
PC2MH	TabNet-2	TabNet-0	***	***	NS	PC2MH	TabNet-2	TabNet-0	***	NS	***
	TabNet-2	ResNet	***	NS	***		TabNet-2	ResNet	***	***	NS
	TabNet-2	RF	***	NS	***		TabNet-2	RF	***	NS	***
	TabNet-2	TabNet-1	***	***	NS		TabNet-2	TabNet-1	***	NS	***
	TabNet-2	MLP	***	NS	***		TabNet-2	MLP	***	***	NS
	TabNet-2	FTT	***	NS	***		TabNet-2	FTT	***	***	NS
	TabNet-2	Ensemble	***	NS	***		TabNet-2	Ensemble	***	***	NS
	TabNet-2	XGBoost	***	NS	***		TabNet-2	XGBoost	***	***	NS
	TabNet-0	ResNet	***	NS	***		TabNet-0	ResNet	***	***	NS
	TabNet-0	RF	***	NS	***		TabNet-0	RF	**	**	NS
	TabNet-0	TabNet-1	***	NS	***		TabNet-0	TabNet-1	***	***	NS
	TabNet-0	MLP	***	NS	***		TabNet-0	MLP	***	***	NS
	TabNet-0	FTT	***	NS	***		TabNet-0	FTT	***	***	NS
	TabNet-0	Ensemble	***	NS	***		TabNet-0	Ensemble	***	***	NS
	TabNet-0	XGBoost	***	NS	***		TabNet-0	XGBoost	***	***	NS
	ResNet	RF	***	***	NS		ResNet	RF	***	NS	***
	ResNet	TabNet-1	***	***	NS		ResNet	TabNet-1	***	NS	***
	ResNet	MLP	***	NS	***		ResNet	MLP	***	NS	***
	ResNet	FTT	***	NS	***		ResNet	FTT	NS	NS	NS
	ResNet	Ensemble	***	NS	***		ResNet	Ensemble	*	*	NS
	ResNet	XGBoost	***	NS	***		ResNet	XGBoost	NS	NS	NS
	RF	TabNet-1	***	***	NS		RF	TabNet-1	NS	NS	NS
	RF	MLP	***	NS	***		RF	MLP	***	***	NS
	RF	FTT	***	NS	***		RF	FTT	***	***	NS
	RF	Ensemble	***	NS	***		RF	Ensemble	***	***	NS
	RF	XGBoost	***	NS	***		RF	XGBoost	***	***	NS
	TabNet-1	MLP	***	NS	***		TabNet-1	MLP	***	***	NS
	TabNet-1	FTT	***	NS	***		TabNet-1	FTT	***	***	NS
	TabNet-1	Ensemble	***	NS	***		TabNet-1	Ensemble	***	***	NS
	TabNet-1	XGBoost	***	NS	***		TabNet-1	XGBoost	***	***	NS
	MLP	FTT	**	NS	**		MLP	FTT	***	***	NS
	MLP	Ensemble	***	NS	***		MLP	Ensemble	***	***	NS
	MLP	XGBoost	NS	NS	NS		MLP	XGBoost	***	***	NS
	FTT	Ensemble	***	NS	***		FTT	Ensemble	*	*	NS
	FTT	XGBoost	**	**	NS		FTT	XGBoost	NS	NS	NS
	Ensemble	XGBoost	***	***	NS		Ensemble	XGBoost	NS	NS	NS

Supplementary Table 5: Bootstrap significance pairwise comparisons between models for Specificity and Sensitivity for the PC2MH task. * $p < 0.05$, ** $p < 0.01$, *** $p < 0.001$, NS = not significant.

Pairwise Model Comparisons for PC2MH Task – AUC & PPV

AUC						PPV					
**Task**	**Model A**	**Model B**	**p**	**p (>)**	**p (<)**	**Task**	**Model A**	**Model B**	**p**	**p (>)**	**p (<)**
PC2MH	TabNet-2	TabNet-0	**	NS	**	PC2MH	TabNet-2	TabNet-0	NS	NS	NS
	TabNet-2	ResNet	***	***	NS		TabNet-2	ResNet	***	NS	***
	TabNet-2	RF	***	NS	***		TabNet-2	RF	***	NS	***
	TabNet-2	TabNet-1	NS	NS	NS		TabNet-2	TabNet-1	*	NS	*
	TabNet-2	MLP	***	***	NS		TabNet-2	MLP	***	NS	***
	TabNet-2	FTT	NS	NS	NS		TabNet-2	FTT	**	NS	**
	TabNet-2	Ensemble	***	NS	***		TabNet-2	Ensemble	NS	NS	NS
	TabNet-2	XGBoost	***	NS	***		TabNet-2	XGBoost	**	NS	**
	TabNet-0	ResNet	***	***	NS		TabNet-0	ResNet	***	NS	***
	TabNet-0	RF	NS	NS	NS		TabNet-0	RF	***	NS	***
	TabNet-0	TabNet-1	***	***	NS		TabNet-0	TabNet-1	*	NS	**
	TabNet-0	MLP	***	***	NS		TabNet-0	MLP	***	NS	***
	TabNet-0	FTT	**	**	NS		TabNet-0	FTT	**	NS	**
	TabNet-0	Ensemble	**	NS	**		TabNet-0	Ensemble	NS	NS	NS
	TabNet-0	XGBoost	NS	NS	NS		TabNet-0	XGBoost	**	NS	**
	ResNet	RF	***	NS	***		ResNet	RF	*	**	NS
	ResNet	TabNet-1	***	NS	***		ResNet	TabNet-1	***	***	NS
	ResNet	MLP	NS	NS	NS		ResNet	MLP	***	NS	***
	ResNet	FTT	***	NS	***		ResNet	FTT	NS	NS	NS
	ResNet	Ensemble	***	NS	***		ResNet	Ensemble	NS	NS	NS
	ResNet	XGBoost	***	NS	***		ResNet	XGBoost	NS	NS	NS
	RF	TabNet-1	***	***	NS		RF	TabNet-1	***	***	NS
	RF	MLP	***	***	NS		RF	MLP	***	NS	***
	RF	FTT	**	**	NS		RF	FTT	**	NS	**
	RF	Ensemble	*	NS	*		RF	Ensemble	NS	NS	NS
	RF	XGBoost	NS	NS	NS		RF	XGBoost	*	NS	*
	TabNet-1	MLP	***	***	NS		TabNet-1	MLP	***	NS	***
	TabNet-1	FTT	NS	NS	NS		TabNet-1	FTT	**	NS	**
	TabNet-1	Ensemble	***	NS	***		TabNet-1	Ensemble	NS	NS	NS
	TabNet-1	XGBoost	***	NS	***		TabNet-1	XGBoost	**	NS	**
	MLP	FTT	***	NS	***		MLP	FTT	*	*	NS
	MLP	Ensemble	***	NS	***		MLP	Ensemble	NS	NS	NS
	MLP	XGBoost	***	NS	***		MLP	XGBoost	***	***	NS
	FTT	Ensemble	***	NS	***		FTT	Ensemble	NS	NS	NS
	FTT	XGBoost	***	NS	***		FTT	XGBoost	NS	NS	NS
	Ensemble	XGBoost	**	**	NS		Ensemble	XGBoost	NS	NS	NS

Supplementary Table 6: Bootstrap significance pairwise comparisons between models for AUC and PPV for the PC2MH task. * $p < 0.05$, ** $p < 0.01$, *** $p < 0.001$, NS = not significant.

Pairwise Model Comparisons for MH2PC Task – Specificity & Sensitivity

Specificity						Sensitivity					
**Task**	**Model A**	**Model B**	**p**	**p(>)**	**p(<)**	**Task**	**Model A**	**Model B**	**p**	**p(>)**	**p(<)**
MH2PC	MLP	RF	***	***	NS		MLP	RF	***	NS	***
	MLP	FTT	***	***	NS		MLP	FTT	***	NS	***
	MLP	TabNet-1	***	***	NS		MLP	TabNet-1	*	NS	**
	MLP	ResNet	***	***	NS		MLP	ResNet	NS	NS	NS
	MLP	Ensemble	*	NS	*		MLP	Ensemble	NS	NS	NS
	MLP	TabNet-0	***	***	NS		MLP	TabNet-0	***	NS	***
	MLP	TabNet-2	***	***	NS		MLP	TabNet-2	***	NS	***
	MLP	XGBoost	**	**	NS		MLP	XGBoost	NS	NS	NS
	RF	FTT	***	NS	***		RF	FTT	***	***	NS
	RF	TabNet-1	***	NS	***		RF	TabNet-1	***	***	NS
	RF	ResNet	***	NS	***		RF	ResNet	***	***	NS
	RF	Ensemble	***	NS	***		RF	Ensemble	***	***	NS
	RF	TabNet-0	***	NS	***		RF	TabNet-0	***	***	NS
	RF	TabNet-2	**	NS	**		RF	TabNet-2	***	***	NS
	RF	XGBoost	***	NS	***		RF	XGBoost	***	***	NS
	FTT	TabNet-1	***	***	NS		FTT	TabNet-1	NS	NS	NS
	FTT	ResNet	***	NS	***		FTT	ResNet	***	***	NS
	FTT	Ensemble	***	NS	***		FTT	Ensemble	***	***	NS
	FTT	TabNet-0	***	***	NS		FTT	TabNet-0	NS	NS	NS
	FTT	TabNet-2	***	***	NS		FTT	TabNet-2	***	NS	***
	FTT	XGBoost	***	NS	***		FTT	XGBoost	***	***	NS
	TabNet-1	ResNet	***	NS	***		TabNet-1	ResNet	NS	*	NS
	TabNet-1	Ensemble	***	NS	***		TabNet-1	Ensemble	***	***	NS
	TabNet-1	TabNet-0	***	***	NS		TabNet-1	TabNet-0	**	NS	**
	TabNet-1	TabNet-2	***	***	NS		TabNet-1	TabNet-2	***	NS	***
	TabNet-1	XGBoost	***	NS	***		TabNet-1	XGBoost	***	***	NS
	ResNet	Ensemble	***	NS	***		ResNet	Ensemble	NS	NS	NS
	ResNet	TabNet-0	***	***	NS		ResNet	TabNet-0	***	NS	***
	ResNet	TabNet-2	***	***	NS		ResNet	TabNet-2	***	NS	***
	ResNet	XGBoost	NS	NS	*		ResNet	XGBoost	NS	NS	NS
	Ensemble	TabNet-0	***	***	NS		Ensemble	TabNet-0	***	NS	***
	Ensemble	TabNet-2	***	***	NS		Ensemble	TabNet-2	***	NS	***
	Ensemble	XGBoost	***	***	NS		Ensemble	XGBoost	NS	NS	NS
	TabNet-0	TabNet-2	***	***	NS		TabNet-0	TabNet-2	NS	NS	*
	TabNet-0	XGBoost	***	NS	***		TabNet-0	XGBoost	***	***	NS
	TabNet-2	XGBoost	***	NS	***		TabNet-2	XGBoost	***	***	NS

Supplementary Table 7: Bootstrap significance pairwise comparisons between models for Specificity and Sensitivity for the MH2PC task. * $p < 0.05$; ** $p < 0.01$; *** $p < 0.001$; NS not significant.

Pairwise Model Comparisons for MH2PC Task – AUC & PPV

AUC						PPV					
**Task**	**Model A**	**Model B**	**p**	**p(>)**	**p(<)**	**Task**	**Model A**	**Model B**	**p**	**p(>)**	**p(<)**
MH2PC	MLP	RF	***	NS	***		MLP	RF	NS	NS	NS
	MLP	FTT	***	NS	***		MLP	FTT	NS	NS	NS
	MLP	TabNet-1	***	NS	***		MLP	TabNet-1	NS	NS	NS
	MLP	ResNet	***	NS	***		MLP	ResNet	NS	NS	NS
	MLP	Ensemble	***	NS	***		MLP	Ensemble	NS	NS	NS
	MLP	TabNet-0	***	NS	***		MLP	TabNet-0	NS	NS	NS
	MLP	TabNet-2	***	NS	***		MLP	TabNet-2	NS	NS	NS
	MLP	XGBoost	***	NS	***		MLP	XGBoost	NS	NS	NS
	RF	FTT	***	***	NS		RF	FTT	***	NS	***
	RF	TabNet-1	***	***	NS		RF	TabNet-1	NS	NS	NS
	RF	ResNet	***	***	NS		RF	ResNet	NS	NS	NS
	RF	Ensemble	NS	NS	NS		RF	Ensemble	***	***	NS
	RF	TabNet-0	*	**	NS		RF	TabNet-0	NS	NS	NS
	RF	TabNet-2	***	***	NS		RF	TabNet-2	***	***	NS
	RF	XGBoost	NS	NS	*		RF	XGBoost	***	***	NS
	FTT	TabNet-1	NS	NS	NS		FTT	TabNet-1	***	***	NS
	FTT	ResNet	***	***	NS		FTT	ResNet	NS	NS	NS
	FTT	Ensemble	***	NS	***		FTT	Ensemble	***	***	NS
	FTT	TabNet-0	*	NS	*		FTT	TabNet-0	***	***	NS
	FTT	TabNet-2	NS	NS	NS		FTT	TabNet-2	***	***	NS
	FTT	XGBoost	***	NS	***		FTT	XGBoost	***	***	NS
	TabNet-1	ResNet	***	***	NS		TabNet-1	ResNet	NS	NS	NS
	TabNet-1	Ensemble	***	NS	***		TabNet-1	Ensemble	***	***	NS
	TabNet-1	TabNet-0	NS	NS	NS		TabNet-1	TabNet-0	NS	NS	*
	TabNet-1	TabNet-2	NS	NS	NS		TabNet-1	TabNet-2	NS	NS	NS
	TabNet-1	XGBoost	***	NS	***		TabNet-1	XGBoost	***	***	NS
	ResNet	Ensemble	***	NS	***		ResNet	Ensemble	NS	NS	NS
	ResNet	TabNet-0	***	NS	***		ResNet	TabNet-0	NS	NS	NS
	ResNet	TabNet-2	***	NS	***		ResNet	TabNet-2	NS	NS	NS
	ResNet	XGBoost	***	NS	***		ResNet	XGBoost	NS	NS	NS
	Ensemble	TabNet-0	*	**	NS		Ensemble	TabNet-0	***	NS	***
	Ensemble	TabNet-2	***	***	NS		Ensemble	TabNet-2	***	NS	***
	Ensemble	XGBoost	NS	NS	NS		Ensemble	XGBoost	NS	NS	NS
	TabNet-0	TabNet-2	NS	NS	NS		TabNet-0	TabNet-2	**	**	NS
	TabNet-0	XGBoost	***	NS	***		TabNet-0	XGBoost	***	***	NS
	TabNet-2	XGBoost	***	NS	***		TabNet-2	XGBoost	***	***	NS

Supplementary Table 8: Bootstrap significance pairwise comparisons between models for AUC and PPV for the MH2PC task. * $p < 0.05$; ** $p < 0.01$; *** $p < 0.001$; NS not significant.

	Primary Care		Mental Health Specialty	
	Attempt	No Attempt	Attempt	No Attempt
All	151	112,394	249	151,570
Race				
White	120	96,447	227	126,368
Black	1	3,697	12	6,093
Other	30	12,081	10	18,687
Ethnicity				
Hispanic	13	12,057	26	20,316
Not Hispanic	138	100,337	223	131,254
Sex				
Male	49	43,003	118	55,519
Female	102	69,391	131	96,051

Supplementary Table 9: Group-wise Attempt and No Attempt counts for test set demographic subgroups of race, ethnicity, and sex, and all data.

Task	Model Winner (AUC)	<u>AUC</u>	PPV	Specificity	Sensitivity
pc2pc	XGBoost	$\mathbf{0.80}_{[0.75,0.84]}$	$0.47_{[0.36,0.59]}$	$1.0_{[1.0,1.0]}$	$0.22_{[0.15,0.29]}$
mh2mh	Random Forest	$\mathbf{0.98}_{[0.97,0.99]}$	$0.94_{[0.91,0.97]}$	$1.0_{[1.0,1.0]}$	$0.82_{[0.78,0.87]}$
mh2pc	XGBoost	$\mathbf{0.79}_{[0.75,0.83]}$	$0.0_{[0.0,0.0]}$	$1.0_{[1.0,1.0]}$	$0.0_{[0.0,0.0]}$
pc2mh	XGBoost	$\mathbf{0.81}_{[0.77,0.84]}$	$0.10_{[0.03,0.17]}$	$1.0_{[1.0,1.0]}$	$0.02_{[0.01,0.04]}$

Supplementary Table 10: Best-performing model per task based on AUC. Mean value reported over 1000 bootstrap samples of the test set with 95% CI in subscript.

Task	Model Winner (Sens.)	AUC	PPV	Specificity	Sensitivity
pc2pc	TabNet-1	$0.73_{[0.67,0.78]}$	$0.01_{[0.01,0.01]}$	$0.94_{[0.94,0.94]}$	$\mathbf{0.44}_{[0.36,0.52]}$
mh2mh	TabNet-1	$0.97_{[0.96,0.99]}$	$0.14_{[0.14,0.15]}$	$0.99_{[0.99,0.99]}$	$\mathbf{0.91}_{[0.88,0.94]}$
mh2pc	Random Forest	$0.77_{[0.72,0.81]}$	$0.03_{[0.02,0.03]}$	$0.98_{[0.98,0.98]}$	$\mathbf{0.35}_{[0.27,0.42]}$
pc2mh	TabNet-0	$0.80_{[0.76,0.83]}$	$0.01_{[0.01,0.01]}$	$0.85_{[0.84,0.85]}$	$\mathbf{0.57}_{[0.51,0.63]}$

Supplementary Table 11: Best-performing model per task based on Sensitivity. Mean values reported over 1000 bootstrap samples of the test set with 95% CI in subscript.

Source	Target	AUC	PPV	Specificity	Sensitivity
PC	PC	$0.62_{[0.57,0.69]}$	$0.003_{[0.002,0.004]}$	$0.84_{[0.84,0.85]}$	$0.36_{[0.28,0.45]}$
MH	MH	$0.65_{[0.62,0.70]}$	$0.01_{[0.01,0.01]}$	$0.89_{[0.89,0.89]}$	$0.42_{[0.36,0.48]}$
MH	PC	$0.70_{[0.64,0.76]}$	$0.03_{[0.02,0.04]}$	$0.99_{[0.99,0.99]}$	$0.24_{[0.18,0.32]}$
PC	MH	$0.69_{[0.65,0.72]}$	$0.003_{[0.003,0.004]}$	$0.74_{[0.74,0.74]}$	$0.49_{[0.42,0.55]}$

Supplementary Table 12: Race: White

Source	Target	AUC	PPV	Specificity	Sensitivity
PC	PC	$0.61_{[0.59,0.62]}$	$0.0_{[0.0,0.0]}$	$0.92_{[0.91,0.93]}$	$0.0_{[0.0,0.0]}$
MH	MH	$0.52_{[0.38,0.67]}$	$0.003_{[0.0,0.01]}$	$0.89_{[0.88,0.90]}$	$0.17_{[0.0,0.42]}$
MH	PC	$0.80_{[0.78,0.81]}$	$0.0_{[0.0,0.0]}$	$0.99_{[0.98,0.99]}$	$0.0_{[0.0,0.0]}$
PC	MH	$0.70_{[0.52,0.84]}$	$0.003_{[0.001,0.005]}$	$0.75_{[0.74,0.76]}$	$0.41_{[0.17,0.67]}$

Supplementary Table 13: Race: Black

Source	Target	AUC	PPV	Specificity	Sensitivity
PC	PC	$0.66_{[0.56,0.76]}$	$0.01_{[0.003,0.01]}$	$0.86_{[0.85,0.86]}$	$0.36_{[0.20,0.53]}$
MH	MH	$0.56_{[0.39,0.75]}$	$0.002_{[0.0,0.004]}$	$0.94_{[0.93,0.94]}$	$0.20_{[0.0,0.50]}$
MH	PC	$0.90_{[0.82,0.96]}$	$0.01_{[0.0,0.04]}$	$0.99_{[0.99,1.0]}$	$0.03_{[0.0,0.10]}$
PC	MH	$0.79_{[0.59,0.92]}$	$0.001_{[0.001,0.002]}$	$0.74_{[0.73,0.75]}$	$0.71_{[0.40,0.90]}$

Supplementary Table 14: Race: Other

Source	Target	AUC	PPV	Specificity	Sensitivity
PC	PC	$0.66_{[0.60,0.71]}$	$0.003_{[0.002,0.004]}$	$0.82_{[0.81,0.82]}$	$0.40_{[0.30,0.49]}$
MH	MH	$0.66_{[0.61,0.71]}$	$0.005_{[0.004,0.01]}$	$0.88_{[0.88,0.89]}$	$0.41_{[0.33,0.49]}$
MH	PC	$0.76_{[0.69,0.82]}$	$0.04_{[0.02,0.05]}$	$0.99_{[0.99,0.99]}$	$0.17_{[0.10,0.24]}$
PC	MH	$0.66_{[0.60,0.72]}$	$0.003_{[0.002,0.003]}$	$0.74_{[0.74,0.74]}$	$0.53_{[0.44,0.61]}$

Supplementary Table 15: Sex: Female

Source	Target	AUC	PPV	Specificity	Sensitivity
PC	PC	$0.57_{[0.47,0.66]}$	$0.003_{[0.002,0.004]}$	$0.90_{[0.89,0.90]}$	$0.26_{[0.14,0.39]}$
MH	MH	$0.63_{[0.57,0.68]}$	$0.01_{[0.01,0.01]}$	$0.92_{[0.92,0.92]}$	$0.40_{[0.31,0.48]}$
MH	PC	$0.68_{[0.60,0.76]}$	$0.02_{[0.01,0.03]}$	$0.98_{[0.98,0.99]}$	$0.25_{[0.12,0.37]}$
PC	MH	$0.72_{[0.68,0.76]}$	$0.004_{[0.003,0.004]}$	$0.74_{[0.74,0.75]}$	$0.45_{[0.36,0.53]}$

Supplementary Table 16: Sex: Male

Source	Target	AUC	PPV	Specificity	Sensitivity
PC	PC	$0.60_{[0.44,0.76]}$	$0.003_{[0.001,0.005]}$	$0.88_{[0.87,0.88]}$	$0.31_{[0.08,0.54]}$
MH	MH	$0.68_{[0.56,0.80]}$	$0.01_{[0.01,0.01]}$	$0.94_{[0.94,0.94]}$	$0.50_{[0.31,0.69]}$
MH	PC	$0.80_{[0.64,0.93]}$	$0.0_{[0.0,0.0]}$	$0.99_{[0.99,0.99]}$	$0.0_{[0.0,0.0]}$
PC	MH	$0.55_{[0.42,0.67]}$	$0.002_{[0.001,0.003]}$	$0.73_{[0.73,0.74]}$	$0.35_{[0.19,0.54]}$

Supplementary Table 17: Ethnicity: Hispanic

Source	Target	AUC	PPV	Specificity	Sensitivity
PC	PC	$0.64_{[0.58,0.69]}$	$0.003_{[0.003,0.004]}$	$0.84_{[0.84,0.85]}$	$0.36_{[0.29,0.44]}$
MH	MH	$0.64_{[0.60,0.68]}$	$0.01_{[0.01,0.01]}$	$0.89_{[0.89,0.89]}$	$0.39_{[0.33,0.45]}$
MH	PC	$0.73_{[0.68,0.78]}$	$0.03_{[0.02,0.04]}$	$0.99_{[0.99,0.99]}$	$0.21_{[0.14,0.28]}$
PC	MH	$0.71_{[0.67,0.74]}$	$0.003_{[0.003,0.004]}$	$0.74_{[0.74,0.75]}$	$0.51_{[0.44,0.57]}$

Supplementary Table 18: Ethnicity: Not Hispanic

	**Demographic Parity Ratio**	**Equalized Odds Ratio**
Race PC2PC (Tab1)	0.492	0.0
Race MH2MH (Tab1)	0.595	0.394
Race PC2MH (Tab0)	0.973	0.595
Race MH2PC (RF)	0.446	0.0
Sex PC2PC (Tab1)	0.569	0.569
Sex MH2MH (Tab1)	0.687	0.683
Sex PC2MH (Tab0)	0.996	0.853
Sex MH2PC (RF)	0.427	0.419
Ethnicity PC2PC (Tab1)	0.791	0.791
Ethnicity MH2MH (Tab1)	0.550	0.547
Ethnicity PC2MH (Tab0)	0.967	0.683
Ethnicity MH2PC (RF)	0.677	0.0

Supplementary Table 19: Fairness Quantification Metrics

	Primary Care		Mental Health Specialty	
Unique Features	Included	Excluded	Included	Excluded
charlson_cpd		×	✓	
benzo_rx_pre5y_cumulative		×	✓	
anx_dx_pre5y_noi_cumulative		×		×
medicaid	✓			×
q9_0_de	✓		✓	
eat_dx_pre5y		×		×
coll_deg_lt25p	✓		✓	
dep_dx_pre5y_noi_cumulative	✓			×
any_inj_poi_pre5y_cumulative	✓		✓	
per_dx_pre5y_noi_cumulative		×		×
antidep_rx_pre3m	✓		✓	
any_sui_att_pre5y_cumulative_a	✓		✓	
phqnumber365	✓			×
mh_ip_pre5y_cumulative	✓		✓	
alc_dx_pre5y_noi_cumulative		×		×
dep_dx_pre5y	✓		✓	
any_sui_att_pre1y_cumulative	✓		✓	
age	✓		✓	
ac1	✓		✓	
charlson_a	✓			×
mh_op_pre5y_cumulative	✓		✓	
any_sui_att_pre3m	✓		✓	
antidep_rx_pre5y_cumulative		×	✓	
phq8_missing_f		×		×
anx_dx_pre5y_cumulative	✓		✓	
mh_op_pre1y_cumulative	✓		✓	
per_dx_pre5y		×		×
ac2		×		×
bip_dx_pre5y_cumulative_f	✓			×
mh_ed_pre5y_cumulative	✓		✓	
commercial	✓		✓	
del_pre_1_365		×		×
anx_dx_pre5y_cumulative_a	✓		✓	

Supplementary Table 20: 33 unique features identified across best ML (XGBoost) and DL (TabNet-1) model for both target tasks (PC and MH). Checkmark means that that unique feature was included in the MHRN identified feature set, and x means it was not. Inclusion for PC is out of the 102 features MHRN identified using LASSO and 94 for MH.

**Unique Feature**	**Description**
charlson_cpd	Charlson comorbidity score for chronic pulmonary disease
benzo_rx_pre5y_cumulative	Cumulative benzodiazepine prescriptions in past 0 to 5 years
anx_dx_pre5y_noi_cumulative	Cumulative anxiety diagnoses (no index, doesn't include info from index encounter) past 0 to 5 years
medicaid	Medicaid insurance indicator
q9_0_de	PHQ-9 question 9 response is 0 and a depression diagnosis in past 0 to 5 years
eat_dx_pre5y	Eating disorder diagnosis in past 1 to 5 years
coll_deg_lt25p	Neighborhood education with </>25% college degree attainment
dep_dx_pre5y_noi_cumulative	Cumulative depression diagnoses (no index, doesn't include info from index encounter) past 0 to 5 years
any_inj_poi_pre5y_cumulative	Any injury/poisoning-related suicide event in past 0 to 5 years
per_dx_pre5y_noi_cumulative	Personality disorder diagnoses (no index, doesn't include info from index encounter) past 0 to 5 years
antidep_rx_pre3m	Antidepressant prescription in past 0 to 3 months
any_sui_att_pre5y_cumulative_a	Any suicide attempt recorded in past 0 to 5 years, interaction with age
phqnumber365	Total number of PHQs in prior 365 days
mh_ip_pre5y_cumulative	Inpatient mental health care in past 0 to 5 years
alc_dx_pre5y_noi_cumulative	Alcohol use disorder diagnosis (no index, doesn't include info from index encounter) past 0 to 5 years
dep_dx_pre5y	Depression diagnosis in past 1 to 5 years
any_sui_att_pre1y_cumulative	Any suicide attempt recorded in past 0 to 1 year
age	Patient age
ac1	Age 13-17
charlson_a	Charlson comorbidity score on index date, interaction with age
mh_op_pre5y_cumulative	Outpatient mental health utilization in past 0 to 5 years
any_sui_att_pre3m	Any suicide attempt recorded in past 0 to 3 months
antidep_rx_pre5y_cumulative	Cumulative antidepressant prescriptions in past 0 to 5 years
phq8_missing_f	PHQ8 missing at visit (1 if missing), interaction with gender=female
anx_dx_pre5y_cumulative	Cumulative anxiety diagnoses in past 0 to 5 years
mh_op_pre1y_cumulative	Outpatient mental health utilization in past 0 to 1 year
per_dx_pre5y	Personality disorder diagnosis in past 1 to 5 years
ac2	Age 18-29
bip_dx_pre5y_cumulative_f	Bipolar disorder diagnosis in past 0 to 5 years, interaction with gender=female
mh_ed_pre5y_cumulative	Emergency department mental health utilization past 0 to 5 years
commercial	Commercial insurance indicator
del_pre_1_365	Delivery in prior 1–365 days of index visit
anx_dx_pre5y_cumulative_a	Anxiety diagnoses in past 0 to 5 years, interaction with age

Supplementary Table 21: Descriptions for 33 unique features used in ML and DL models.
